# Characterization of novel loci controlling seed oil content in *Brassica napus* by marker metabolite-based multi-omics analysis

**DOI:** 10.1186/s13059-023-02984-z

**Published:** 2023-06-19

**Authors:** Long Li, Zhitao Tian, Jie Chen, Zengdong Tan, Yuting Zhang, Hu Zhao, Xiaowei Wu, Xuan Yao, Weiwei Wen, Wei Chen, Liang Guo

**Affiliations:** 1 National Key Laboratory of Crop Genetic Improvement, Huazhong Agricultural University, Wuhan, China; 2 Hubei Hongshan Laboratory, Wuhan, China; 3 Key Laboratory of Horticultural Plant Biology (MOE), College of Horticulture and Forestry Sciences, Huazhong Agricultural University, Wuhan, China; 4 Shenzhen Institute of Nutrition and Health, Huazhong Agricultural University, Wuhan, China; 5 Shenzhen Branch, Guangdong Laboratory for Lingnan Modern Agriculture, Genome Analysis Laboratory of the Ministry of Agriculture, Agricultural Genomics Institute at Shenzhen, Chinese Academy of Agricultural Sciences, Shenzhen, China

**Keywords:** *Brassica napus*, Metabolomics, mGWAS, mTWAS, WGCNA, Seed oil content

## Abstract

**Background:**

Seed oil content is an important agronomic trait of *Brassica napus* (*B. napus*), and metabolites are considered as the bridge between genotype and phenotype for physical traits.

**Results:**

Using a widely targeted metabolomics analysis in a natural population of 388 *B. napus* inbred lines, we quantify 2172 metabolites in mature seeds by liquid chromatography mass spectrometry, in which 131 marker metabolites are identified to be correlated with seed oil content. These metabolites are then selected for further metabolite genome-wide association study and metabolite transcriptome-wide association study. Combined with weighted correlation network analysis, we construct a triple relationship network, which includes 21,000 edges and 4384 nodes among metabolites, metabolite quantitative trait loci, genes, and co-expression modules. We validate the function of BnaA03.TT4, BnaC02.TT4, and BnaC05.UK, three candidate genes predicted by multi-omics analysis, which show significant impacts on seed oil content through regulating flavonoid metabolism in *B. napus*.

**Conclusions:**

This study demonstrates the advantage of utilizing marker metabolites integrated with multi-omics analysis to dissect the genetic basis of agronomic traits in crops.

**Supplementary Information:**

The online version contains supplementary material available at 10.1186/s13059-023-02984-z.

## Background

The plant kingdom contributes the most metabolite diversity and produces 100,000 to 1 million types of metabolites [1]. Plant metabolites are essential for plant growth and development, and they play key roles in the defense and adaptation to the environment [2, 3]. Metabolites link genotype to phenotype for physical traits [4–6]. Over recent years, more and more studies have discovered the connection between marker metabolites and agronomic traits, such as primary metabolites and starch-, cold sweetening-related traits in potato [7]; fruit load in tomato [8], lignin precursors, and biomass in maize [9]; and secondary metabolites and maize kernel weight [10]. Compared with conventional QTL mapping of agronomic traits which usually discovers a limited quantity of loci [11–14], secondary metabolites have a highly polygenic architecture for large-effect loci [10, 15–18]. A vast array of studies shows that metabolomics is a powerful tool for grasping a better understanding of plant metabolite synthesis and functions [10, 15, 19, 20].

Metabolite genome-wide association studies (mGWAS) have detected lots of loci that are related to corresponding metabolites in several main crops, such as rice [15, 21], maize [10, 18], and wheat [22]. Transcriptome-wide association studies (TWAS) have been developed to interpret the relationship between gene expression and phenotype with whole-genome gene expression levels taken into account [23, 24]. Recent studies indicate that TWAS has been a novel and powerful tool for gene-phenotype prediction [25–27]. Metabolite TWAS (mTWAS) can unearth more metabolite-associated and trait-correlated genes. Expression genome-wide association study (eGWAS) is an approach using gene expression information to perform GWAS [28]. Therefore, gene expression diversity can be explained by genomic variation, and the expression quantitative trait loci (eQTLs) can imply the potential regulatory network of genes for dissecting complex traits [29, 30]. High-throughput metabolomics analysis united with multi-omics analysis is powerful for dissecting the genetic basis of complex traits in crops.

*Brassica napus* is the world’s third-largest oil crop with a roughly 13% production of edible oil around the world. Improvement of seed oil content (SOC) has been one of the most important goals for *B. napus* breeding [31–33]. The first step of fatty acid (FA) synthesis in plants occurs in the plastid [34], where the carboxylation of acetyl-CoA is catalyzed by acetyl-CoA carboxylase to produce malonyl-CoA [35–37]. Then, fatty acid synthase uses malonyl-CoA as a substrate, adding two carbons per cycle to synthesize acyl carbon chains to synthesize saturated fatty acids with 16 and 18 carbons [38]. Furthermore, malonyl-CoA is also a substrate for flavonoid biosynthesis. Many genes are responsible for flavonoid biosynthesis and regulation in plants [39–41]. TRANSPARENT TESTA 4 (TT4) catalyzes the conversion of malonyl-CoA and 4-coumaroyl-CoA to chalcone in the first step of the flavonoid biosynthesis pathway [42, 43]. Mutation of *TT4* leads to a dramatic decrease in flavonoids and about 14% increase in SOC in *Arabidopsis* [43]. It has also been reported that knockout of *TT2* or *TT8* in *B. napus* using CRISPR/Cas9 technology results in a yellow-seeded phenotype with a significant increase in oil content and a significant decrease in flavonoids in seeds [44–46]. Yellow-seeded seed has less seed coat content (SCC), higher meal protein content, and higher SOC than black-seeded seed [47, 48], and there is a huge economic value for yellow-seeded *B. napus* breeding.

In this study, a comprehensive metabolome blueprint has been drawn in *B. napus* seed. By integrating multi-omics data including genome, transcriptome, and metabolome, we dissected the genetic basis of SOC in *B. napus* by performing mGWAS, mTWAS, eGWAS, and WGCNA analysis. We identified 131 SOC-correlated marker metabolites and identified 445 metabolite quantitative trait loci (mQTL) of these marker metabolites. SOC-correlated modules were subsequently characterized, and these results indicate the potential regulation relationships between genes and metabolites. Finally, we experimentally validated three candidate genes (*BnaA03.TT4*, *BnaC02.TT4*, and *BnaC05.UK*), which impact SOC through the regulation of metabolome in the seeds.

## Results

### Metabolic profiling constructs a metabolome landscape of *B. napus* seed

**Fig. 1:**
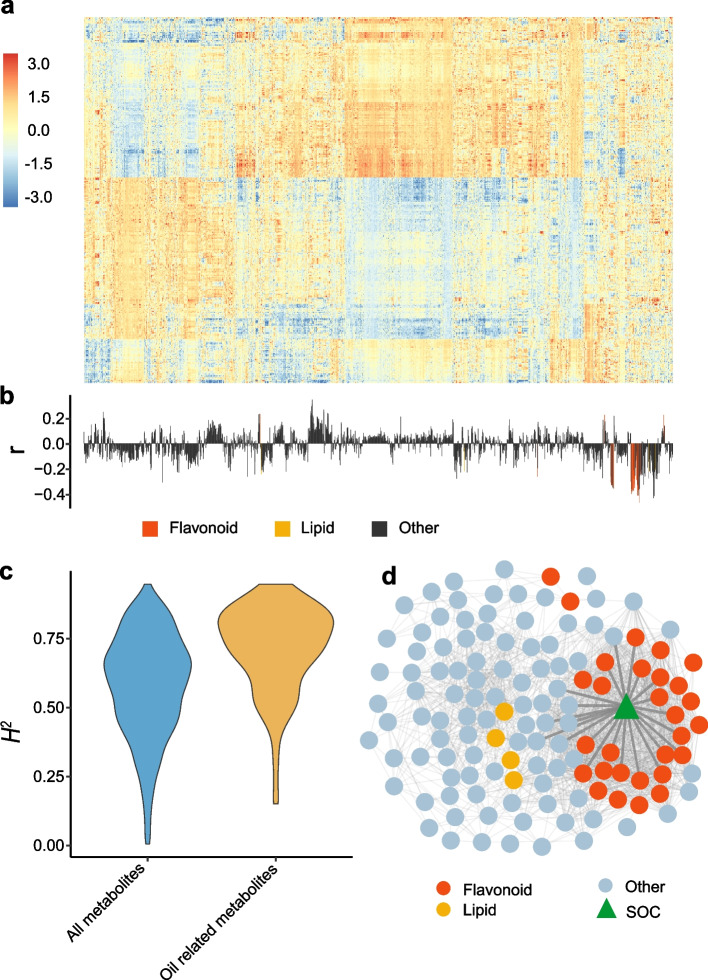
Profiling of metabolites in the mature seed of a *B. napus* natural population. **a** The heatmap of 2173 detected metabolites in the natural population. The horizontal axis represents the metabolites detected in each individual, and the vertical axis shows the individuals. The color of each cell represents the normalized content of the metabolite in the related individual. **b** The correlation between the detected metabolites and SOC. Each vertical line represents a metabolite that corresponds to the above column in **a**. **c** Distribution of the values of broad-sense heritability (*H*^2^) of 2173 metabolites and 131 SOC-correlated metabolites (*p* = 2.2 × 10^−16^). **d** Network of metabolites-metabolites and SOC-metabolites (|*r*|> 0.35), the connections between metabolites and SOC are bolded

A total of 2173 metabolites, including 80 amino acid derivatives, 38 nucleic acid derivatives, 60 lipids, 92 flavonoids, 29 phenylethanoids, 42 terpenoids, and 99 other metabolites, have been quantified in the mature seeds of 388 *B. napus* accessions using the widely targeted metabolomics method (Fig. 1a; Additional file 1: Table S1 and S2) [19], and the metabolite content data in 2 years are listed in Additional file 1: Table S3 and Table S4. To investigate the metabolic changes affecting the SOC in *B. napus*, 131 SOC-correlated metabolites (|*r*|> 0.2) are identified as marker metabolites of SOC, including 26 positively correlated metabolites and 105 negatively correlated ones (Fig. 1b), which are selected for further analysis. From the heatmap of the relative content of all metabolites, we find that most of those SOC-correlated metabolites exhibit similar accumulation patterns (Fig. 1a, b). Besides, we have calculated the broad-sense heritability of all metabolites, showing a wide variation range from 0.01 to 0.95 with a mean value of 0.58, and the SOC-correlated metabolites have a higher mean value of 0.71 (Fig. 1c). These results suggest that elevated heritability in SOC correlated metabolites by biasing the samples towards SOC variation. We have annotated 45 SOC-correlated metabolites based on standard and reference spectral library, and 30 metabolites are annotated as flavonoids and 4 metabolites are annotated as lipids (Additional file 1: Table S5). Most (27 out of 30) of the flavonoids and all 4 lipids (|*r*|> 0.20) are negatively correlated with SOC (Additional file 1: Table S5, Additional file 2: Fig. S1). In addition, a hypergeometric test was conducted to rule out the possible bias of significant correlation between the SOC and the flavonoid family metabolites (*P* = 6.75 × 10^−16^, Additional file 1: Table S6). For better studying more correlated SOC markers, we use a higher threshold (|*r*|> 0.35) and find that flavonoids are more correlated to SOC than other metabolites (Fig. 1d). These results suggest that they can serve as marker metabolites of SOC in *B. napus*.

### Association analysis between metabolome, transcriptome, and variome uncovers mQTLs potentially affecting SOC

**Fig. 2:**
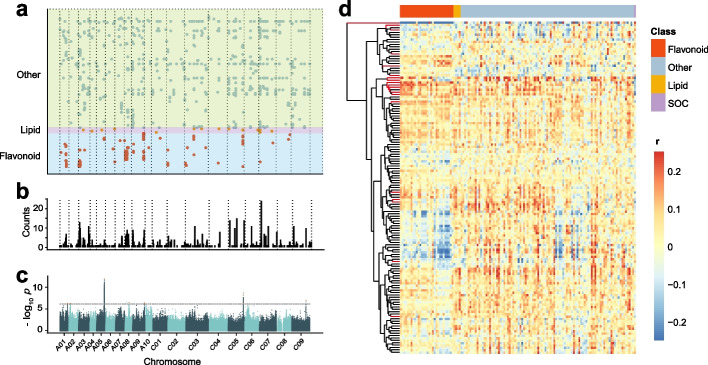
mGWAS and module-trait association studies of SOC and SOC-correlated metabolites. **a** Chromosomal distribution of all lead SNPs associated with SOC-correlated metabolites (the results of 2017 and 2018 are combined). **b** Chromosomal distribution of all mQTLs associated with SOC-correlated metabolites (the results of 2017 and 2018 are combined). The interval size is 100 kb. **c** Manhattan plot of GWAS results of the BLUP values of SOC across 5 years in WH [25]. **d** Heatmap of correlation among SOC-correlated metabolites, SOC, and eigenvalues of modules obtained by WGCNA. Each eigenvalue represents the profile of the genes in the correlated module. The vertical axis shows the modules, and the branch in red represents the modules significantly correlated with SOC

A total number of 8,274,830 and 8,288,519 variations (minor allele frequency > 5%) in 388 *B. napus* accessions have been selected for conducting mGWAS for SOC-correlated metabolites in 2 years respectively (2017 and 2018) [25]. A Bonferroni correction of *p* = 1.2 × 10^−7^ which is described in the methods has been employed as the genome-wide threshold for 131 SOC-correlated metabolites, and a total of 1318 variation-metabolite associations for 128 metabolites are detected, including 329, 17, and 972 associations corresponding to 29 flavonoids, 4 lipids, and 95 other metabolites, respectively (Fig. 2a; Additional file 1: Table S7). All the signals can be divided into 445 mQTLs according to the LD blocks of this panel (100 kb for each block) [25], and there are obvious hotspots in the association analysis. Twelve mQTLs on chromosomes A03, C03, C05, C06, C07, and C09 are detected by more than 10 SOC-correlated metabolites (Fig. 2b; Additional file 1: Table S7). In total, six mQTLs detected by SOC-correlated metabolites are colocalized with SOC-related loci detected previously [25] (Fig. 2c; Table 1).

**Table 1:** Summary of the hotspot loci detected by mGWAS co-localized with SOC-related loci [25]

mQTL^a^	Chromosome	Position	Metabolites^b^	Environment (SOC)^c^
182	A01	21,217,005–21,240,857	S21_S-4234	W3/H4/H6/A6
422	A03	2,231,384–2,368,373	S21_S-0148, S21_S-0470, S21_S-1706, S21_S-3662, S21_S-0649, S21_S-0968, S21_S-3600, S21_L-0232, S21_S-1022, S21_S-2354, S21_S-3675	W4/H5/H6/A6
956	A05	20,294,478–20,328,897	mr1077, mr1203, S21-4865	W1/W2/W3/W4/W5/W6/H4/H5/A6/H6
1507	A08	10,480,586–10,489,644	S21-1083, S21-1235	W2/W6/A6
1863	A09	31,177,191–31,257,536	S21_L-0942, mr1103, mr1226, mr1227, mr1228, mr1082, mr1083	W2/W6/A6
3947	C05	39,949,109–40,027,927	S21-1612, wm0013, mr4076, wm0031, mr002, mr1263, S21-4865, mr1204, S21_S-1264, S21-4990, mr4064	W1/W2/W3/W4/W6/C4/A6

^a^The IDs of the QTL detected by SOC-correlated metabolites

^b^The IDs of the SOC metabolites

^c^Previous GWAS study on SOC of *B. napus* [25]. Environments in this study. WH, W; HF, H; CD, C; 2013, 1; 2014, 2; 2016, 3; 2017, 4; 2018, 5; BLUP-predicted oil content in WH, W6; BLUP-predicted oil content in HF, H6; BLUP-predicted oil content in three locations, A6

In our previous study, we obtained the population transcriptome data 40 days after flowering (DAF) developing seeds [25]. To better understand the linkage between transcripts and SOC-correlated metabolites, we have performed mTWAS using the 40 DAF transcriptome dataset with 70,781 genes. mTWAS identifies 7316 genes having significant association with SOC-correlated metabolites (*p* < 1.41 × 10^−5^; Additional file 1: Table S8). WGCNA has been conducted for all these transcripts associated with SOC-correlated metabolites, which are clustered into 139 modules (Additional file 1: Table S9), and the correlation coefficient of module eigengenes to SOC-correlated metabolites and SOC are then calculated (Fig. 2d; Additional file 1: Table S10). Among all of the modules, 19 modules are significantly correlated with SOC, especially module 15 (size = 946, *r* = 0.41, *p* = 6.58 × 10^−11^). The GO enrichment analysis indicates that module 15 is enriched in the flavonoid biosynthetic and long-chain fatty-acyl-CoA metabolic processes (Additional file 2: Fig. S2a). In addition, module 27, module 43, and module 136 are also enriched in the lipid metabolic and seed development processes (Additional file 2: Fig. S2b-S2d).

**Fig. 3:**
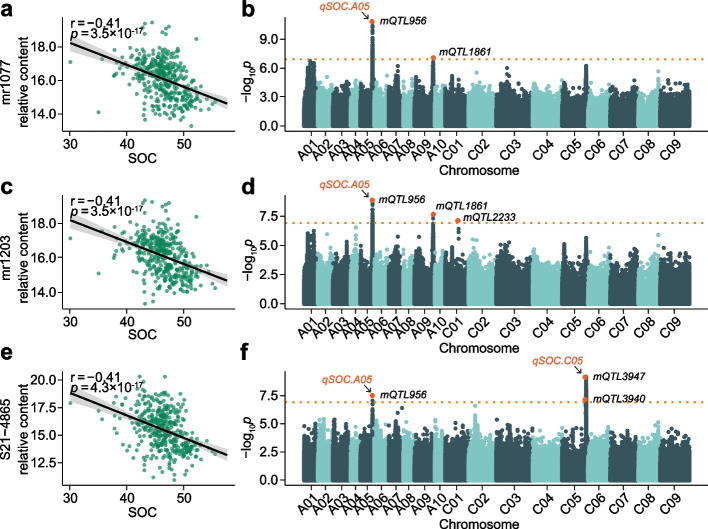
mGWAS results related to the loci on chromosome A05 (position = 20,328,269 bp) (2017). **a** Correlation analysis between SOC and mr1077 (Chrysoeriol *C*-hexoside). **b** Manhattan plot of mGWAS for mr1077. **c** Correlation analysis between SOC and mr1203 (methylluteolin *C*-hexoside). **d** Manhattan plot of mGWAS for mr1203. **e** Correlation analysis between SOC and S21-4865. **f** Manhattan plot of mGWAS for S21-4865. *qSOC.A05* and *qSOC.C05* are two stable SOC-related QTLs identified in our previous study [25]

GWAS for SOC suggests a significant associative peak (*qSOC.A05*) on chromosome A05 (position = 20,328,269, *p* = 2.64 × 10^−8^) which is also identified in our previous study [25]. Interestingly, this locus is also detected by Chrysoeriol *C*-hexoside (lead SNP *p* = 1.83 × 10^−11^), methylLuteolin *C*-hexoside (lead SNP *p* = 1.37 × 10^−9^), and an unknown metabolite (lead SNP *p* = 2.85 × 10^−8^) (Additional file 1: Table S7). These three metabolites are negatively correlated with SOC (Fig. 3a–f). In addition, it is interesting that a SOC-related locus is detected on chromosome A09 (position = 31,257,536, *p* = 4.04 × 10^−7^), and this position is also co-localized with a hot block detected by 7 SOC-correlated flavonoids (Additional file 2: Fig. S3a-3n). According to the above six co-localized loci, six lead SNPs are used to train a random forest model for predicting the SOC levels. The SOC level of each training and testing set is calculated based on the SOC under five environments. The training set including 108 low SOC individuals (range from 29.4 to 37.8%), 189 medium SOC individuals (range from 37.6 to 40.6%), and 55 high SOC individuals (range from 41.0 to 47.6%) is utilized to train the model (Additional file 2: Fig. S4a-S4c). The test set includes 47 low SOC individuals, 82 medium SOC individuals, and 23 high SOC individuals which are utilized for testing the predictive accuracy of the models trained by the lead SNPs and random SNPs. The test area under curves (AUCs) for the model trained with the peak SNPs are 0.61 for the low SOC, 0.54 for the medium SOC, and 0.72 for the high SOC individuals. The mean AUCs for the model trained with the random SNPs are 0.52 for the low SOC, 0.51 for the medium SOC, and 0.52 for the high SOC individuals (Additional file 2: Fig. S4d-S4f). The results indicate that these six peak SNPs could be used for marker-assisted selection of high SOC individuals, and these findings will facilitate the future *B. napus* breeding.

To uncover the relationship between genomic polymorphism and gene expression, 7316 genes associated with SOC-correlated metabolites are selected for eQTL calculation in this panel (Additional file 1: Tables S8, S11). As a result, 117,782 lead SNPs are significantly associated with the selected genes, and those SNPs are united into 5242 eQTLs, if their physical distance is < 100 kb (Additional file 1: Table S8). Among all the eQTLs, some eQTLs on chromosomes A03, A08, A09, and C09 are associated with more than 200 genes (Additional file 2: Fig. S5). It suggests that there might be master regulators in those eQTLs to regulate a set of genes related to SOC-correlated metabolites, directly or/and indirectly influencing SOC.

### Triple relationship network discovers promising genes for improving SOC

**Fig. 4:**
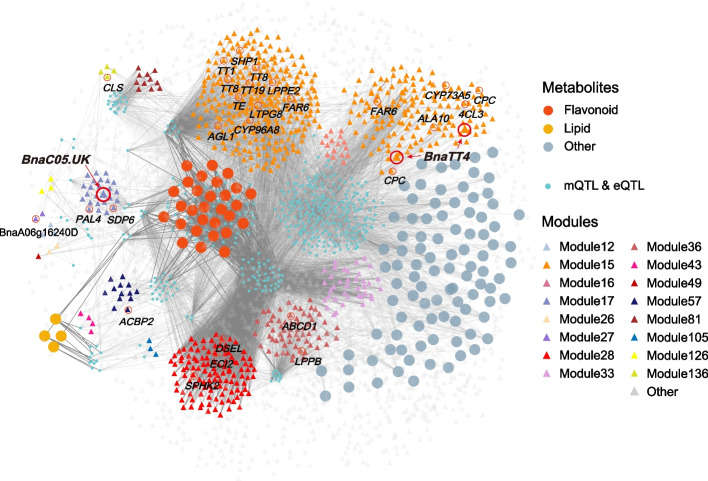
Network built based on the correlation among SOC-correlated metabolites, genes, and QTLs. Genes are shown as reverse arrows with a distinct color per co-expression module. All modules significantly correlated with SOC were shown in this network, except for module 68, module 134, and module 135. The QTLs were detected simultaneously by the significant associated genes (eQTL) and metabolites (mQTL) in this triple relationship network

To gain a full comprehension of the genome, transcriptome, and variome, the mGWAS results and transcriptome analysis results have been integrated into a triple relationship network (metabolite-QTL-gene) (Fig. 4; Additional file 1: Table S12), which includes 444 QTLs identified simultaneously by both metabolites and transcripts, 3813 genes, and 127 SOC correlated metabolites. According to the triple relationship network and the results of mTWAS, we have selected 240 candidate genes based on gene functional annotation, which may be related to SOC accumulation (Additional file 1: Table S13). For example, Shatterproof (*SHP1*) is a MADS-BOX transcript factor (TF), along with *SHP2* control the lignification of dehiscence zone cells in plants [49]. *SHP1* and *SHP2* are essential for ovule development [50]. In our study, the transcript level of *BnaA04.SHP1* (BnaA04g01810D) is significantly negatively correlated with SOC (*r* =  − 0.32, *p* = 2.2 × 10^−7^, Additional file 2: Fig. S6a). Interestingly, mTWAS results indicate that *BnaA04.SHP1* is significantly associated with 44 metabolites negatively correlated with SOC (Additional file 1: Table S13). eGWAS suggests the expression level of *BnaA04.SHP1* is associated with two *cis*-eQTLs and six *trans*-eQTLs (Additional file 2: Fig. S6b-S6c). Haplotype analysis shows that haplotype N has a higher expression level of *BnaA04.SHP1* (*p* = 2.0 × 10^−11^, Additional file 2: Fig. S6d). In addition, CAPRICE (*CPC*) is an MYB TF, which positively regulates the root development [51] and represses the biosynthesis of anthocyanin in plants [52]. In our study, the transcript level of *BnaA05.CPC* (BnaA05g01400D) is negatively correlated with SOC (*r* =  − 0.15, *p* = 0.015, Additional file 2: Fig. S7a). mTWAS results showed that *BnaA05.CPC* is significantly associated with 33 SOC-correlated metabolites (Additional file 1: Table S13). eGWAS suggests the expression level of *BnaA05.CPC* is associated with one *cis*-eQTL and five *trans*-eQTLs (Additional file 2: Fig. S7b-S7c). Haplotype analysis shows that haplotype T has a higher expression level of *BnaA05.CPC* (*p* = 8.2 × 10^−9^, Additional file 2: Fig. S7d). It indicates that there are potential regulation networks of *BnaA04.SHP1* and *BnaA05.CPC* individually, and these two genes could be responsible for the biosynthesis of flavonoids and influencing SOC in *B. napus*.

### *BnaTT4* negatively impacts SOC as a key enzyme of flavonoid biosynthesis in *B. napus* seed

**Fig. 5:**
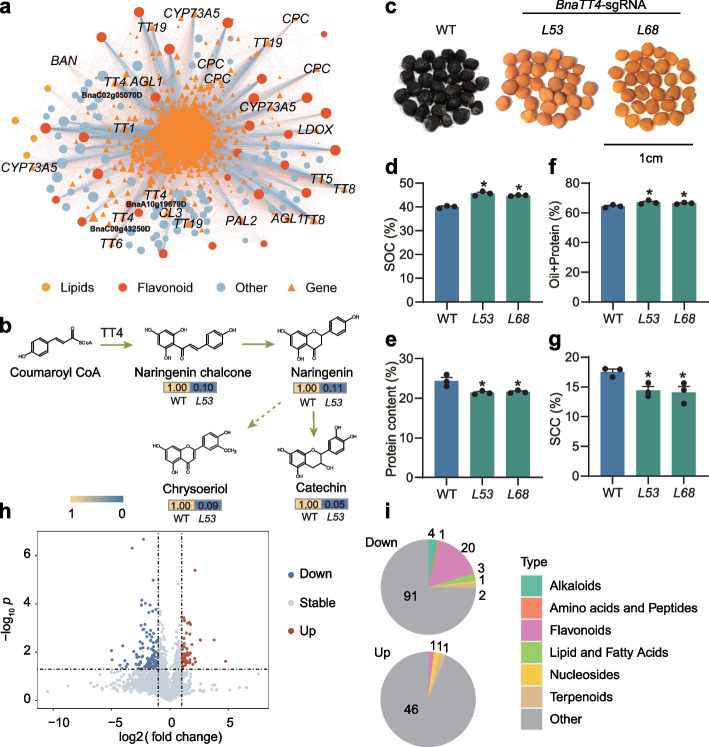
Functional identification of *BnaTT4* as a positive regulator of seed flavonoid biosynthesis in *B. napus*. **a** Pearson correlation of genes in module 15 with SOC-correlated metabolites (|*r*|> 0.40). The absolute values of Pearson’s correlation coefficient values are represented by the transparency of lines. **b** Accumulation level of metabolites in a flavonoid reaction chain of WT and *Bnatt4* line. **c** Seeds of CRISPR/Cas9-induced *BnaTT4* homozygous lines (*L53* and *L68*). Bar = 1 cm. **d** SOC in the *BnaTT4* knockout mutant seeds and WT seeds. **e** Protein content in the *BnaTT4* knockout mutant seeds and WT seeds. **f** Oil and protein content in the *BnaTT4* knockout mutant seeds and WT seeds. **g** SCC in the *BnaTT4* knockout mutant seeds and WT seeds. **h** The volcano plot for differentially accumulated metabolites in *Bnatt4* line vs WT (FC > 2 and adjusted *p* < 0.05). The red and blue points indicate up- and downregulated metabolites, respectively. **i** Number of up- and downregulated metabolites and their classification. Values are means ± s.e.m., *n* = 3. Statistical analysis is using Student’s *t*-test (*p < 0.05)

Chalcone synthase (TRANSPARENT TESTA 4, TT4) is the first step reaction of flavonoid biosynthesis [53]. mGWAS for mr1600 (luteolin *C*-sinapoyl hexoside) identifies a significant SNP on chromosome A03 (*mQTL421*, position = 2,231,384, *p* = 7.39 × 10^−14^, Additional file 1: Table S7). We find *BnaA03.TT4* (BnaA03g04590D) is in the mQTL region (Additional file 2: Fig. S8a-S8b). Similarly, mGWAS for mr1976 suggests a significant SNP on chromosome C02 (*mQTL2396*, position = 2,740,593, *p* = 1.16 × 10^−8^, Additional file 1: Table S7) and *BnaC02.TT4* (BnaC02g05070D) is likely the candidate gene (Additional file 2: Fig. S8c-S8d). mTWAS analysis suggests that three *BnaTT4* homologs are significantly associated with over 40 metabolites, and the majority of the associated metabolites are flavonoids (Additional file 2: Fig. S8e). *BnaTT4* homologous genes in *B. napus* show significant correlations with SOC and flavonoid metabolites in our population, especially BnaC09g43250D, BnaA10g19670D, and BnaC02g05070D are also included in module 15 (Figs. 4 and 5a; Additional file 2: Figs. S9a and S9b). Thus, we validated the function of *BnaTT4* using mutants generated by CRISPR/Cas9, which is homozygous mutants (T_2_) of BnaA02g30340D, BnaC02g05070D, BnaA03g04590D, BnaC03g06120D, BnaA10g19670D, and BnaC09g43250D (Additional file 1: Table S14). Naringenin chalcone is the direct production of TT4, which is dramatically decreased in the *BnaTT4* mutant, as well as naringenin and Chrysoeriol (Fig. 5b). *BnTT4* mutant lines not only have yellow-seeded phenotype (Fig. 5c) as expected but also show a higher SOC increased by 5.6% (*L53*) and 4.8% (*L68*) than wild type (WT) (Fig. 5d). Although *BnaTT4* mutants have a decrease in protein content, the total percentage of oil and protein is significantly increased in both mutants (Fig. 5e, f). Moreover, *BnaTT4* mutant lines show a significantly lower SCC than WT (Fig. 5g). In addition, we have analyzed the metabolome changes of *L53*, and there are 122 decreased metabolites and 49 increased metabolites (Fig. 5h). Among the known metabolites, most of the decreased metabolites are flavonoids and alkaloids (Fig. 5i). For example, catechin (mr002, SOC *r* =  − 0.47) is one of the metabolites most significantly correlated to SOC in our dataset. It is significantly associated with five *BnaTT4*s in mTWAS (BnaC02g05070D, *p* = 5.59 × 10^−14^; BnaC09g43250D, *p* = 8.72 × 10^−12^; BnaA10g19670D, *p* = 5.46 × 10^−8^; BnaA03g04590D, *p* = 5.79 × 10^−6^; BnaC03g06120D, *p* = 9.29 × 10^−6^, Additional file 1: Table S13). Compared to WT, catechin is barely existent in *L53* (*Bnatt4*/WT = 0.05, Fig. 5b). Similarly, Chrysoeriol is barely existent in the mutants (*Bnatt4*/WT = 0.09, Fig. 5b), almost all the detected flavonoids exhibit a similar pattern. We have surveyed *BnaTT4s* mutant growth phenotypes, except for the thousand seed weight is significantly decreased, we have not observed any yield penalties (Additional file 1: Table S15). The results suggest that the synthesis of flavonoids is blocked in the *BnaTT4s* mutants and the carbon source may shift to seed oil synthesis when *BnaTT4s* are interrupted.

### *BnaC05.UK* is a novel gene negatively impacting SOC in *B. napus*

**Fig. 6:**
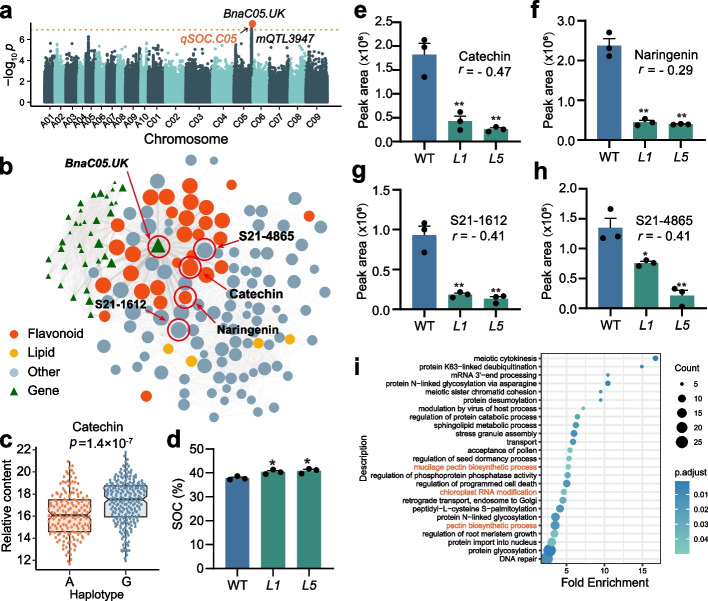
Functional study of *BnaC05.UK*. **a** Manhattan plot of mGWAS results of mr002 (2017). **b** Association analysis of genes in module 17 with SOC-correlated metabolites (|*r*|> 0.30). The absolute values of Pearson’s correlation coefficient values are represented by the thickness of lines. **c** Haplotype analysis of *mQTL3947* for mr002 content. **d** SOC of WT and mutant seeds. **e** Peak area of mr002, **f** naringenin, **g** S21-1612, and **h** S21-4865 in WT and mutant seeds. **i** Enrichment analysis for genes that are significantly different between WT and *L5*. Values are means ± s.e.m., *n* = 3. Statistical analysis is using Student’s *t*-test (*p < 0.05; **p < 0.01)

We find an mQTL hotspot with linked variations on chromosome C05, including *mQTL3946* and *mQTL3947*, which are associated with 16 SOC correlated metabolites (Fig. 6a; Additional file 2: Fig. S10a-S10t; Additional file 1: Table S7). Interestingly, this hotspot is co-localized with the SOC-related QTL identified in our previous study [25]. mGWAS for catechin suggests a significant SNP signal on chromosome C05 (Fig. 6a, position = 40,022,989, *p* = 5.66 × 10^−8^). It belongs to the *mQTL3947* which is detected by three flavonoids and is co-localized with an SCC-related QTL detected in our previous study [46] (Additional file 1: Table S7 and Additional file 2: Fig. S11a-S11d). Gene module analysis indicates that module 17 is not only significantly correlated with SOC but also shows significant correlations with lipid and flavonoid metabolites (Fig. 6b; Additional file 1: Table S10). We predicted a gene *BnaC05.UK* (BnaC05g43050D) with unknown biological functions as the candidate gene in *mQTL3947*. Because *BnaC05.UK*’s expression is significantly associated with 9 metabolites including catechin in mTWAS analysis (Additional file 1: Table S8). Importantly, *BnaC05.UK* is one of the core genes in module 17 (Fig. 6b). eGWAS reveals that there is a promoter region variation maybe responsible for the metabolite and SOC variations (Additional file 1: Table S8 and Additional file 2: Fig. S12a-S12c). Moreover *BnaC05.UK* is significantly negatively correlated with SOC (*r* =  − 0.36,* p* = 9.5 × 10^−9^, Additional file 2: Fig. S12d). Haplotype analysis suggests that *BnaC05.UK* with haplotype G represent higher catechin (Fig. 6c, SOC *r* =  − 0.47, *p* = 1.4 × 10^−7^). We built a phylogenetic tree of *BnaC05.UK* using its cDNA sequences. There are seven sequences out of 12 belonging to *Brassica* (Additional file 2: Fig. S12e). In addition, we analyzed the basic information about *BnaC05.UK*, and its open reading frame length is 1348 bp including one exon (276 bp encoding 91 amino acids) in the Darmor genome [54]. We also analyzed the sequence of *BnaC05.UK* in the pangenome of *B napus* in BnTIR [55] (http://yanglab.hzau.edu.cn/BnTIR, Additional file 2: Fig. S12f). There is also no typical domain for *BnaC05.UK* analyzed by InterPro [56] (http://www.ebi.ac.uk/interpro/). Furthermore, there are no studies reporting the function of *BnaC05.UK* or its homologous gene.

Our multi-omics analysis suggests that *BnaC05.UK* plays important functions in affecting the level of many metabolites and may affect SOC in *B. napus*. In order to investigate the function of *BnaC05.UK*, we generated *BnaC05.UK* homozygous mutants (*T*_2_) using *Westar* and the mutation lines show significantly higher SOC of about 2.6% (*L1*) and 3.1% (*L5*) than WT (Fig. 6d; Additional file 1: Table S14). As we mentioned before, *mQTL3947* is detected by mGWAS for 16 metabolites. Metabolome analysis suggests that the level of catechin, naringenin (SOC *r* =  − 0.29), S21-1612 (SOC *r* =  − 0.41), and S21-4865 (SOC *r* =  − 0.41) shows dramatic reduction (Fig. 6e–h). In addition, we performed RNA-seq using WT and *L5* developing seeds (37 DAF), 706 genes were upregulated, and 62 genes are downregulated. Interestingly, differentially expressed genes (DEGs) are enriched in the pectin biosynthetic process, mucilage pectin biosynthetic process, chloroplast RNA modification, and others (Fig. 6i). Among the DEGs, chloroplast RNA modification-related genes are also significantly changed (Additional file 2: Fig. S13a-S13e). In addition, the *Rhamnogalacturonan I rhamnosyltransferase 1* [57], *Probable galacturonosyltransferase 5* [58], and *Probable pectin methyltransferase QUA3* [59] genes have been reported to be involved in the formation of pectin and seed coat mucilage. The expressions of these genes in developing seed of mutation lines are significantly increased (Additional file 2: Fig. S13f-S13k). Three oil accumulation-related genes have increased expression levels (Additional file 2: Fig. S13l-S13n). We have also found that the mutants have more leakage of the seed coat mucilage in mature seeds compared with WT (Additional file 2: Fig. S13o). *BnaC05.UK* is localized in the chloroplast (Additional file 2: Fig. S13p), we speculate that the mutation of this gene may lead to changes in chloroplast RNA levels and affect the accumulation of fatty acids, flavonoids, and pectin [60]. We have also surveyed *BnaC05.UK* mutant growth phenotypes, we have not observed any yield penalties (Additional file 1: Table S16).

## Discussion

Metabolites are considered as important markers for predicting traits, for example, sugar, and amino acid content could predict the accumulation of photosynthate at the final stage [7, 8, 61]. GWAS as well as TWAS have been proven to be efficient tools for genetic dissection [25–27, 62–64]. In our study, we intentionally screened 131 marker metabolites correlated to SOC, and multi-omics analysis efficiently located multiple loci potentially controlling SOC. Many SOC-associated loci (Table 1) were found to be co-localized with the ones located in our previous study [25], showing good reliability and repeatability of these two studies. The association studies and network analysis also provide a reference for future genetic improvement of *B. napus. BnaA05.PMT6* and *BnaC05.PMT6* were successfully cloned from two SOC-related loci *qSOC.A05* and *qSOC.C05* [25]. However, in addition to *BnaC05.PMT6*, we also found *BnaC05.UK* is a candidate gene regulating SOC in *qSOC.C05*, suggesting that there may be two or more genes regulating SOC in *qSOC.C05*. Furthermore, our study confirmed the effect of flavonoid metabolism on SOC, especially catechin and other flavonoid metabolites through functional verification of candidate genes (Figs. 5 and 6). Therefore, our study provided an effective approach for mining marker metabolites and candidate genes for agronomic traits in crops.

The huge metabolite diversity has a powerful role to explore the genetic diversity combined with multi-omics studies, and we discovered novel loci related to SOC by mGWAS. We have shown that SOC-correlated metabolites detected more signals by mGWAS (Fig. 2a–c), which enriches the information on our association population’s genetic diversity for future reference. Using TWAS to dissect traits have been reported in humans [65, 66], animals [67], and plants [20, 26, 27]. In this study, TWAS has been performed using the 40 DAF population transcriptome data of *B. napus*, and united with metabolomics, we propose the concept of mTWAS for the first time. Compared with mGWAS, mTWAS tend to use the expression level of a certain gene, eliminating the uncertainty of linkage disequilibrium interval and screening candidate genes more accurately and directly [68, 69]. Our previous study comprehensively characterized the transcriptional variability in developing seeds of *B. napus* and successfully cloned two transcription factors involved in the regulation of SOC from eQTL hotspots87-88 [70]. The metabolome variation data has brought more information which helped predict novel genes regulating SOC by marker metabolite-based multi-omics analysis. In addition, we have constructed a triple relationship network. Combining co-expression modules and the relationship networks, it is worth looking forward to improving the prediction of crop agronomic traits and assisting crop breeding with marker metabolites in the future.

To validate the results of the meta-analysis, we have functionally analyzed two genes *BnaA03.TT4* and *BnaC02.TT4*, and *BnaTT4s* mutants have increased SOC by 4.8% and 5.6% (Fig. 5d). We found that all 26 flavonoids are associated with *TT4* expression in our mTWAS results (Additional file 1: Table S8). This may be due to the key biological function of TT4 at the first and rate-limiting step in the flavonoid synthesis pathway [43]. These results reflect the effectiveness of our research strategy. We have also identified a novel gene *BnaC05.UK* as a negative regulator of SOC in *B. napus*, because the mutation of *BnaC05.UK* causes a 2.6% and 3.1% increase in SOC (Fig. 6d). *BnaC05.UK* is in the hotspot of metabolites, SOC, and SCC; 16 metabolites are co-localized in *mQTL3947* that are negatively correlated with SOC (Additional file 2: Figs. S10-S11). The content of most of the negative marker metabolites was significantly decreased, explaining the increased SOC in the mutants (Fig. 6e–h). Combined with the transcriptome results of developing mutant seeds, the expression levels of pectin mucilage-related genes were upregulated, and mature mutant seeds have more mucilage leakage than the wild type (Additional file 2: Fig. S13o). The increased SOC and decreased content of flavonoid metabolites in the mutants suggest that *BnaC05.UK* positively regulates flavonoids and negatively regulates oil accumulation. Since flavonoid synthesis is blocked in mutants, excess carbon sources may flow towards fatty acid and seed coat mucilage synthesis. More inspiringly, *BnaC05.UK* is expressed in the chloroplast (Additional file 2: Fig. S13p), where fatty acids are de novo synthesized [38, 71]. The expression of genes related to seed RNA editing in the chloroplast changes significantly during the development stage of the mutant seed, which may cause the disorder of RNA level in the chloroplast [60]. The expression levels of genes related to fatty acid synthesis are also significantly increased in *Palmitoyl-acyl carrier protein thioesterase* [72], *Ethylene-responsive transcription factor ABI4* [73], etc., which is also consistent with the increased SOC phenotype of the mutants (Additional file 2: Fig. S13l-S13n). Pangenome sequence analysis indicates that most of the reference genomes present one homolog of *BnaC05.UK* except for No2127. No2127 is a resynthesized *B. napus* cultivar, and its reference genome presents two homologs (Additional file 2: Fig. S12f). How *UK* gene affects the synthesis of flavonoids, fatty acids, pectin, and the biological significance of their derived evolution needs further study. TT4 catalyzes the first step of flavonoid synthesis; it starts with one molecule of coumaroyl CoA and three molecules of malonyl CoA [43]. In fatty acid synthesis, malonyl CoA is the substrate of fatty acid chain elongation [38]. Fatty acid synthesis and flavonoid synthesis compete with each other for malonyl CoA (Additional file 2: Fig. S14). Published studies have proved that blocking the genes related to flavonoid synthesis will lead to an increase in SOC [44–46]. Of the 30 flavonoids significantly correlated to SOC in this study, 27 flavonoids are significantly negatively correlated to SOC (Additional file 1: Table S5). The mGWAS and mTWAS mapping results of these metabolites also predicted *TT1*, *TT4*, *TT5*, *TT8*, *TT19*, and other known genes to be involved in flavonoid metabolism (Additional file 1: Table S13). Based on multi-omics analysis and using metabolite phenotype as a bridge, we provided direct genetic evidence to confirm the significant negative correlation between oil content and flavonoid synthesis genes (Additional file 1: Table S13). In order to explore the relationship between phenylpropanoids and fatty acid synthesis, we predicted the potential regulatory transcription factors of *BnaTT4s* and *BnaC05.UK* by multi-omics analysis and integrated them into a schematic diagram (Additional file 1: Table S17; Additional file 2: Fig. S14). Behind the simple shuffling carbon phenomenon, it usually exists more complex regulatory relationships within flavonoids/phenolics and SOC, such as *TT2* [74] and *TT8* [75]. Whether *TT4* as a key enzyme participates in a more complexed upstream regulation network needs further investigation.

Although we primarily focused on 131 SOC-correlated metabolites, most of which were flavonoids (Additional file 1: Table S5), and the rest were also worthy of investigation. Thousand seed weight (TSW) is also an important component of *B. napus* yield [76]. It is vital to study the genetic variation of TSW in *B. napus* [77]. Combined with the metabolome map provided here, it is possible to mine more unreported TSW-related genes for breeding. *B. napus* is an important oil-producing crop, and the remaining meal can also be used as animal feed because of its rich protein [47, 48]. Therefore, the research on the protein content of the *B. napus* seeds is also of great value [78, 79]. Our metabolome data included amino acid metabolites, such as L-lysine (mr1330) and L-valine (mr1322) (Additional file 1: Table S5), which will facilitate future research on improvements for the protein content of *B. napus* seeds. In addition, we also quantified many health-related metabolites, such as melatonin (mr2076, Additional file 1: Table S2). It is proven to be effective for insomnia, mood swing, drug abuse, and even cancer treatment [80, 81]. We can unearth certain reliable loci controlling melatonin in *B. napus* [82].

It is worth noting the limitation of our extraction method, which has been previously reported to have excellent analytical confidence for water-soluble metabolites including flavonoids, phenolamines, terpenoids, amino acids, and nucleic acid derivatives [10, 15, 20]. Our study provides rich information on metabolomic diversity in *B. napus* seeds. However, most structures of metabolites remain unknown. Metabolite identification using mass spectrometry tandem chromatography (LC–MS/MS) requires high consistency of experimental conditions [83, 84]. In addition, based on the tight relationship between enzymes and metabolites, the mQTL localization can also be considered as auxiliary information for metabolite analysis [15]. Recently, utilizing software based on machine learning to resolve metabolite structures has become well-welcomed [85–87]. Comprehensive chemical standard identification combined with artificial intelligence annotation will enable large-scale annotation of the metabolome data in the future.

## Conclusions

We performed a metabolomics analysis of *B. napus* mature seeds and obtained 131 SOC-correlated marker metabolites. Based on genome, transcriptome, and metabolome data, we discovered novel genes such as *BnaC05.UK* involved in the regulation of *B. napus* SOC. This study demonstrates the advantage of marker metabolite-based multi-omics analysis to dissect the genetic basis of agronomic traits in crops.

## Methods

### Plant materials and collection of SOC phenotype data

For plant materials, 505 *B. napus* accessions were planted in Wuhan (2017, 2018) [25]. We used Foss NIRSystems 5000 near-infrared reflectance spectroscope that performed mature open-pollinated seed oil content analysis, and each accession was analyzed with 6 replications [88]. Transgenic *B. napus* plants were grown in the field for transgenic crops at Huazhong Agricultural University. For field experiments, we followed a randomized complete block design with three replications.

### Metabolite profiling

A subpopulation was selected for profiling the metabolites in *B. napus* seeds (388 individuals harvested in 2017 and 378 individuals harvested in 2018, Additional file 1: Table S1). For each sample, 0.1 g mature *B. napus* seeds was weighted, and they were homogenized and comminuted by TissueLyser II machine (Qiagen, Germany) at 29 Hz, 60 s with 1 ml 70% methanol containing 0.1 mg/l acyclovir (internal standard). The mixture was vortexed, and the metabolites were extracted for 10 h at 4 °C. After being centrifuged at 9000* g* for 10 min, all the supernatants were collected and then were pooled and filtered with a 0.22-µm organic filter (SCAA-104; ANPEL, Shanghai, China, http://www.anpel.com.cn/) for LC–MS analysis.

A liquid chromatography-electrospray ionization tandem mass spectrometry system was utilized to relatively quantify the metabolites of *B. napus*. In the beginning, an MS^2^ spectral tag (MS2T) library was established using a mixture extraction of 30 accessions, which were selected randomly from the analysis population, following the method described previously [19]. In detail, the MS2T library was established with LIT experiments which were conducted on a triple quadrupole-linear ion trap mass spectrometer (QTRAP), API 4000 Q TRAP LC/MS/MS System, in a positive ion mode and controlled by the Analyst 1.5 software (AB Sciex). In LIT experiments, MIM–EPI was carried out to screen metabolites and restricted the fragments from *m*/*z* 50 to 1000 with a mass step of 0.2 Da. For each feature in the MS2T library, the accurate MS data were acquired with an Agilent 6520 accurate-mass time-of-flight (Q–TOF) mass spectrometry equipped with a dual ESI electrospray ion source in positive ion mode. The features in this MS2T library were annotated using our standards or by comparing the accurate MS data to the reference MS2 library, such as MassBank, HMDB, METLIN, and MzCloud. Quantification of metabolites was carried out using a scheduled multiple reaction monitoring method, and a total of 2173 metabolic features were detected and quantified in the mixture extraction. The 2173 metabolic features of the population were detected, quantified, and then log_2_ transformed and were performed for further normalization.

### mGWAS, mTWAS, eGWAS, and co-expression analysis

For mGWAS and eGWAS, the variants were called by the meta-analysis of reads against the *B. napus* genome (*B. napus* v4.1, http://www.genoscope.cns.fr/brassicanapus/) as described previously [25, 54]. A total of 8,274,830, 8,288,519, 8,365,725, 8,258,336, 8,234,950 variations with minor allele frequency > 5% were selected by PLINK [89] and used to conduct mGWAS for SOC-correlated metabolites content in 2017 and 2018, SOC, SCC, and genes expression with the FaST-LMM software [90]. The significant value was set to 1.2 × 10^−7^ and finally, and the Manhattan plot was generated in the R package. For mTWAS, gene expression of 274 accessions at 40 DAF planted in Wuhan (2017) was called by the meta-analysis of RNA sequencing data, as described previously [25]. Then, the gene expression was used to conduct associative analysis with the content of SOC-correlated metabolites in *B. napus* in the same year by linear regression [91]. The significant value was set to 1/70,781. For co-expression analysis, a total of 70,781 genes were used and analyzed with an R package named WGCNA [92]. Then, all the genes were clustered into 139 modules, with a soft-thresholding power (*β* = 6) to approximate scale-free topology within the network determined.

### Network building for metabolome, transcriptome, and variome

The edges between SOC-correlated metabolites and genetic loci represent the significant signals of mQTLs, while the edges between genetic loci and genes represent the significant signals of eQTLs. Firstly, significant signals for 131 SOC-correlated metabolites were detected with a significant threshold value used, and the significant signals were later clustered into mQTLs according to the LD blocks of this panel (100 kb for each block). Secondly, 7316 genes related to the 131 SOC-correlated metabolites (*p* < 1.41 × 10^−5^) were selected for conducting eGWAS; the significant signals were also clustered into eQTLs according to the LD blocks of this panel (100 kb for each block). Finally, the loci distance was calculated between mQTLs and eQTLs for linking the SOC-correlated metabolites and genes with a threshold of 100 kb utilized.

### Model construction for SOC prediction

To achieve precise SOC classification, the SOC datasets from all five environments (2013, 2014, 2016, 2017, 2018) were collected. The inbred lines (505 individuals) were categorized into three classes based on SOC using *K*-means clustering implemented in PYTHON/SCIKIT-LEARN (*K*-means function, n_clusters = 3, random_state = 2). The inbred lines with high, low, and middling SOC were divided into the training set and testing set (2:1). We identified the peak SNPs according to the results of the mGWAS and co-localized QTLs of metabolites and SOC. To generate robust prediction models, random forest models to predict SOC levels were used (n_estimators = 300, max_features = 3) [93–95]. The training set of six peak SNPs and six random SNPs (100 times) was entered into the random forest models independently to train the prediction models. The area under the curve (AUC) value was calculated to evaluate the accuracy of the model.

### Vector construction and plant transformation

The full-length *BnaC05.UK* CDS was cloned from accession X1072. CRISPR-Cas9 genome editing system [96] were used to generate *tt4* and *uk* mutation lines. PCR fragment and *pKSE401* vector were integrated by Golden Gate Assembly and sequenced for confirmation. Furthermore, the plant transformation of *Westar* was performed with *pKSE401* vectors to generate transgenic lines. The tissue culture method followed Dai et al. [97]. CRISPR mutant lines were comforted by sequencing, and all primers are listed in Additional file 1: Table S18.

### SOC measurement

SOC analysis was performed on near-infrared reflectance spectroscopy with a Foss NIRSystems 5000 near-infrared reflectance spectroscope [88] and GCMS-QP2010 Ultra (SHIMADZU, Inc.) [98] using mature open-pollinated seeds. In brief, about 10 mg seed samples was weighed, adding 3 mL methanol containing 1.5% H_2_SO_4_ and 0.01% butylated hydroxyl toluene (BHT) to the glass tube, then crushed the seeds. Internal standard is heptadecanoic acid (16.2 μmol/mL). To extract and methylate the FA, we performed an 85 °C water bath for 2 h, using 2-mL hexane to extract fatty acid methyl esters (FAMEs). Loading volume is 1 μL, automatic injection. The instrument parameters are as follows: the injection port temperature was 230 °C; oven temperature kept at 170 °C for 1 min, temperature rises gradually (3 °C per minute) and keep at 230 °C for 3 min; and ion source temperature was 200 °C. Calculation of FA is using heptadecanoic acid.

### Subcellular localization

The *BnaC05.UK* coding region was cloned and integrated into the vector pMDC83 to express BnaC05.UK-GFP fusion protein. The primers used are listed in Additional file 1: Table S18. After the recombinant constructs were transformed into *Agrobacterium* strain GV3101, it was infiltrated into tobacco leaves with P19 for transient expression. We observed BnaC05.UK-GFP proteins in epidermal cells using confocal laser scanning microscopy (FV1200, Olympus, Japan).

### Ruthenium red staining of seed-coat mucilage

We followed the method of Li et al. [99]. In general, dry mature seeds were infiltrated with 0.05 mM EDTA (pH 8.5) for 1 h, then stained with 0.01% ruthenium red (w/v) for another 1 h and washed by ddH_2_O. Seed-coat mucilage was photted using an optical microscope (BX53M, Olympus, Japan).

## Supplementary Information

**Additional file 1: Table S1.** Summary of accessions for mGWAS. **Table S2.** The metabolites detected in the study. Table S3. The metabolite content in 2017. **Table S4.** The metabolite content in 2018. **Table S5.** The metabolites correlated to SOC. **Table S6.** Hypergeometric test of the 2,173 metabolites. **Table S7.** mGWAS results of 2 years SOC-correlated metabolites and 2017 SCC. **Table S8.** Summary of significant genes in mTWAS for SOC-correlated metabolites at 40 DAF. **Table S9.** The genes' correlation information of 40 DAF transcriptome. **Table S10.** The statistics of modules calculated by WGCNA. **Table S11.** Information for eQTLs. **Table S12.** The integrated network of mGWAS and eQTL. **Table S13.** The candidate genes associated with SOC from mGWAS , eQTL and mTWAS. **Table S14.** The genotype of BnaTT4 and BnaC05.UK mutant lines in the T2 generation. **Table S15.** The mean value of yield traits for the BnaTT4 CRISPR lines. **Table S16.** The mean value of yield traits for the BnaC05.UK CRISPR lines. **Table S17.** Transcritionnal factors prediction of BnaTT4s and BnaC05.UK. **Table S18.** Primers used in this study.

**Additional file 2: Fig. S1.** Key metabolites structures and mass spectrum plots. **Fig. S2**. Enrichment analysis for genes in modules significantly correlated with SOC. **Fig. S3.** mGWAS results related to the loci on chromosome A09. **Fig. S4**. The performance of metabolite-based Random Forest model for predicting the SOC level. **Fig. S5**. The genome-wide distribution of eQTLs. **Fig. S6**. Genetic analysis of BnaA04g01810D. **Fig. S7**. Genetic analysis of BnaA05g01400D. **Fig. S8.** mGWAS and mTWAS results of BnaTT4s. **Fig. S9**. Correlation analysis of BnaTT4s with SOC and SOC-correlated flavonoids. **Fig. S10**. mGWAS resultsrelated to the loci on chromosome C05. **Fig. S11**. mGWAS resultsrelated to SCC and mr002. **Fig. S12.** Functional study of *BnaC05.UK*. **Fig. S13.** The relative expression of differentially expressed genes between WT and *L5. ***Fig. S14.** Phenylpropanoids and fatty acid synthesis relationship and predicted transcriptional regulation of BnaTT4s and BnaC05.UK.

**Additional file 3. **Review History.

## Data Availability

The genome resequencing data of 382 *B. napus* inbreed lines and gene expression data of developing seeds (40 DAF) of 274 inbreed lines are available under Genome Sequence Archive (GSA; https://bigd.big.ac.cn/gsa/) with Bioproject ID PRJCA002835 [100] and PRJCA002836 [101]. Mass spectrometry data of seed metabolome of 382 *B. napus* inbreed lines are deposited at China National Center for Bioinformation (CNCB; https://www.cncb.ac.cn) with Bioproject ID of PRJCA017446 [102]. No other scripts and software were used other than those mentioned in the “ Methods” section. All the materials in this study are available upon request.
