# Supplementary material for: Characterization of novel loci controlling seed oil content in Brassica napus by marker metabolite-based multi-omics analysis
Source: Genome Biol. 2023 Jun 19;24:141. doi: 10.1186/s13059-023-02984-z (PMC10278308; doi:10.1186/s13059-023-02984-z)
Supplement: Supplementary file 2 — Additional file 2: Fig. S1. Key metabolites structures and mass spectrum plots. Fig. S2. Enrichment analysis for genes in modules significantly correlated with SOC. Fig. S3. mGWAS results related to the loci on chromosome A09. Fig. S4. The performance of metabolite-based Random Forest model for predicting the SOC level. Fig. S5. The genome-wide distribution of eQTLs. Fig. S6. Genetic analysis of BnaA04g01810D. Fig. S7. Genetic analysis of BnaA05g01400D. Fig. S8. mGWAS and mTWAS results of BnaTT4s. Fig. S9. Correlation analysis of BnaTT4s with SOC and SOC-correlated flavonoids. Fig. S10. mGWAS resultsrelated to the loci on chromosome C05. Fig. S11. mGWAS resultsrelated to SCC and mr002. Fig. S12. Functional study of BnaC05.UK. Fig. S13. The relative expression of differentially expressed genes between WT and L5. Fig. S14. Phenylpropanoids and fatty acid synthesis relationship and predicted transcriptional regulation of BnaTT4s and BnaC05.UK. [file 13059_2023_2984_MOESM2_ESM.pdf]

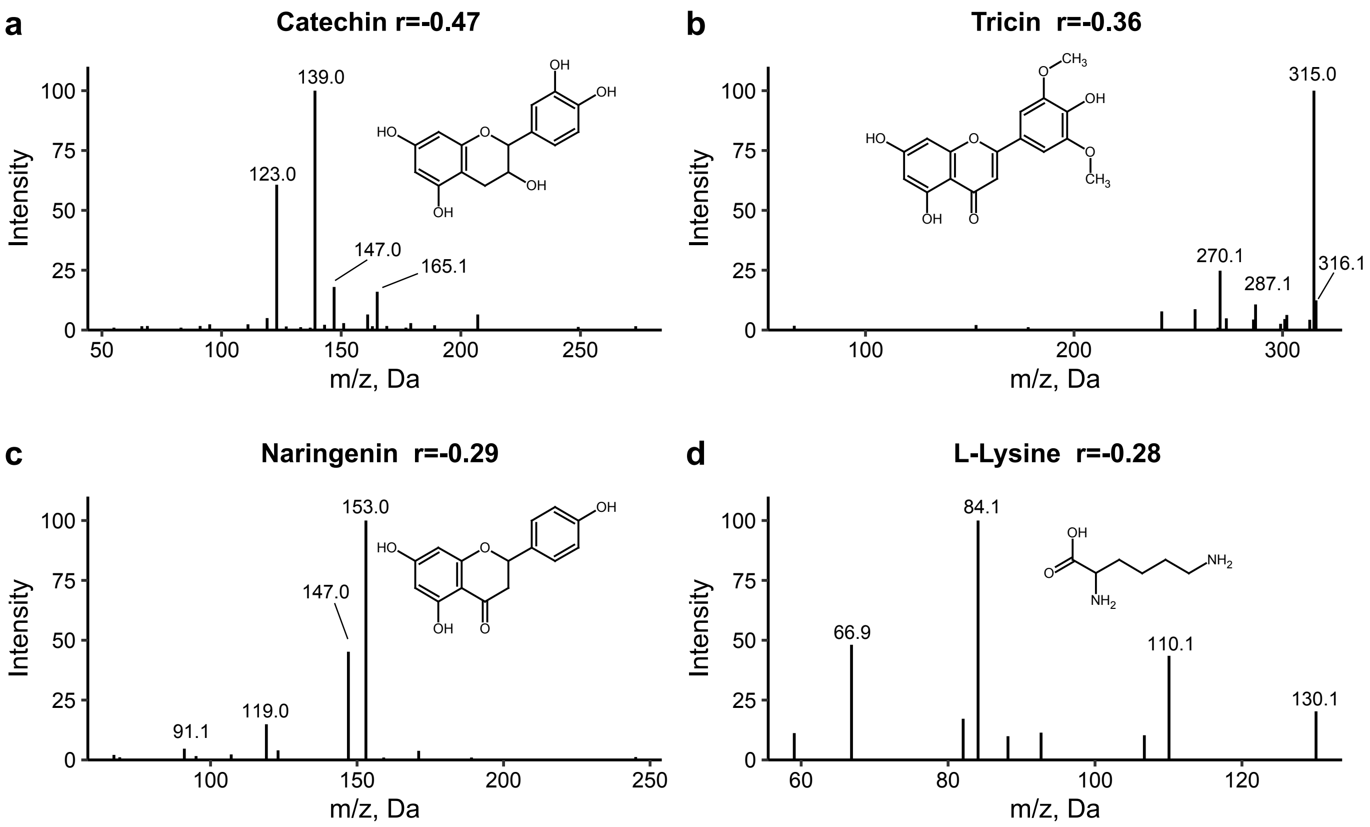

Fig. S1 Key metabolites structures and mass spectrum plots.
a mr002. **b** mr1058. **c** mr1263. **d** mr1330.

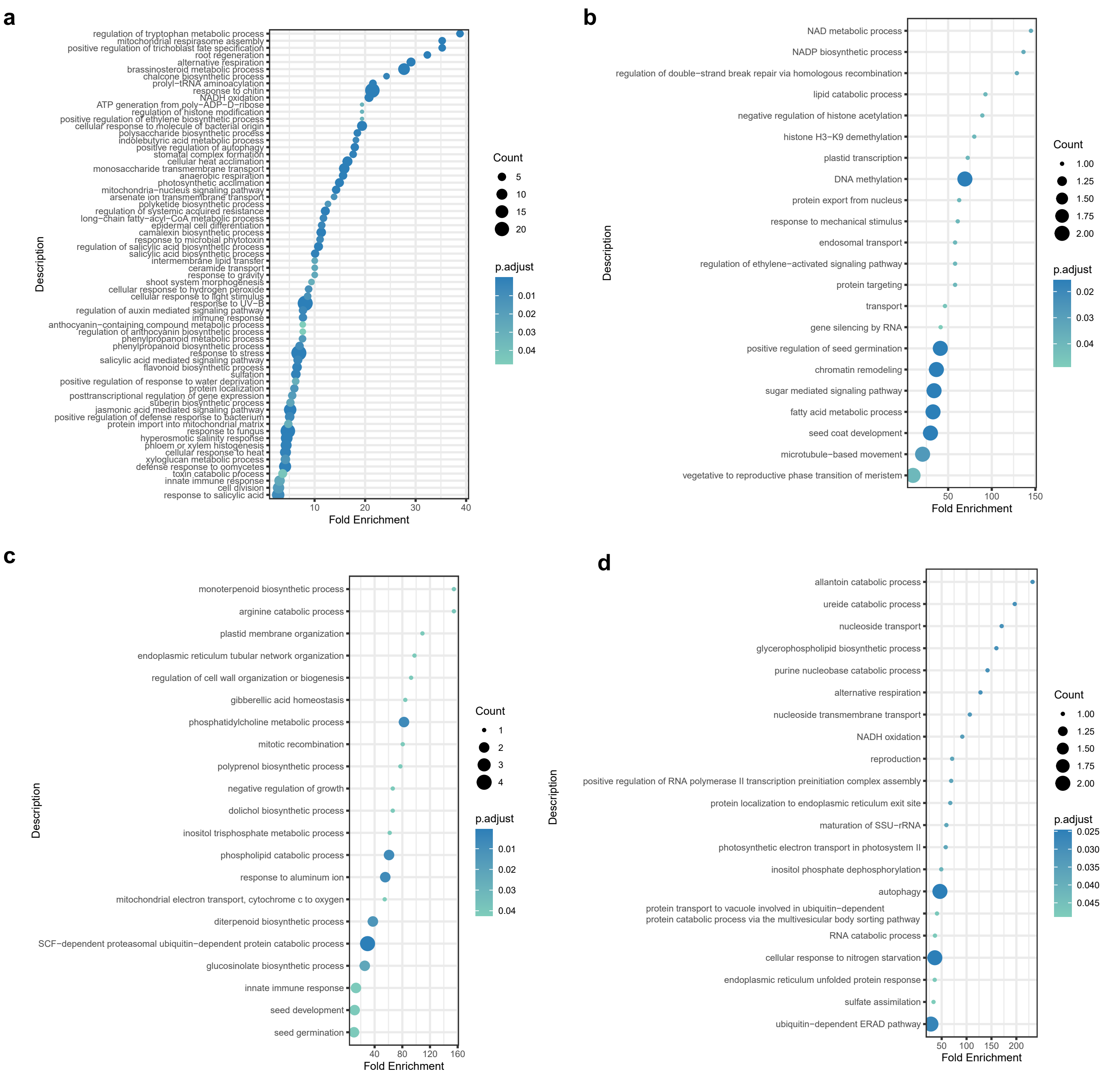

Fig. S2 Enrichment analysis for genes in modules significantly correlated with SOC.

a Enrichment analysis for genes in Module 15. **b** Enrichment analysis for genes in Module 27. **c** Enrichment analysis for genes in Module 43. **d** Enrichment analysis for genes in Module 136.

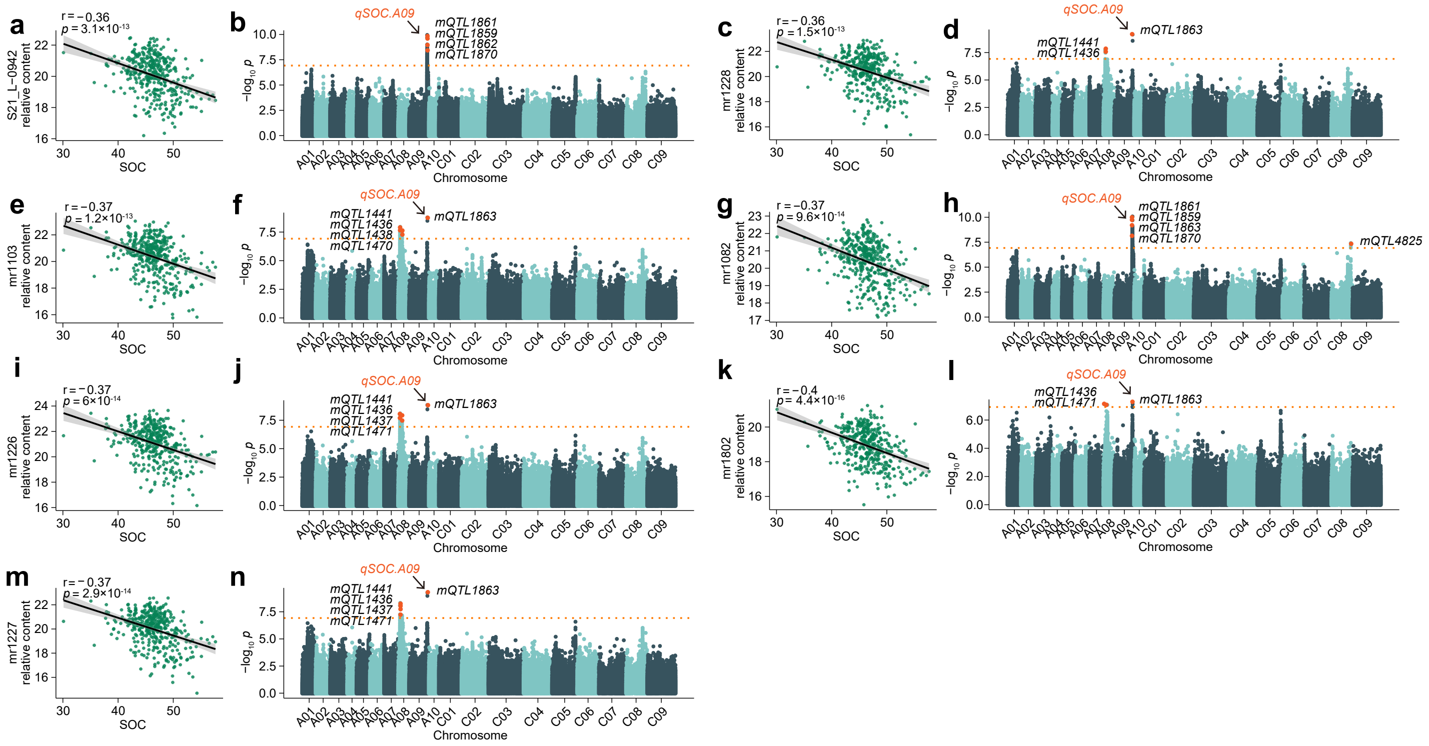

Fig. S3 mGWAS results related to the loci on chromosome A09 (position = 31,257,536 bp) (2017). **a** Correlation analysis between SOC and S21_L-0942 (Isorhamnetin 5-*O*-glucoside). **b** Manhattan plot of mGWAS for S21_L-0942. **c** Correlation analysis between SOC and mr1103 (Tricin 7-*O*-hexosyl-*O*-hexoside). **d** Manhattan plot of mGWAS for mr1103. **e** Correlation analysis between SOC and mr1226 (Tricin 5-*O*-hexosyl-*O*-hexoside). **f** Manhattan plot of mGWAS for mr1226. **g** Correlation analysis between SOC and mr1227 (Tricin 5-*O*-hexosyl-7-*O*-hexoside). **h** Manhattan plot of mGWAS for mr1227. **i** Correlation analysis between SOC and mr1228 (Tricin *O*-hexosyl-*O*-hexoside derivative). **j** Manhattan plot of mGWAS for mr1228. **k** Correlation analysis between SOC and mr1082 (Isorhamnetin 7-*O*-glucoside). **l** Manhattan plot of mGWAS for mr1082. **m** Correlation analysis between SOC and mr1083 (Tricin 5-*O*-hexoside). **n** Manhattan plot of mGWAS for mr1083.

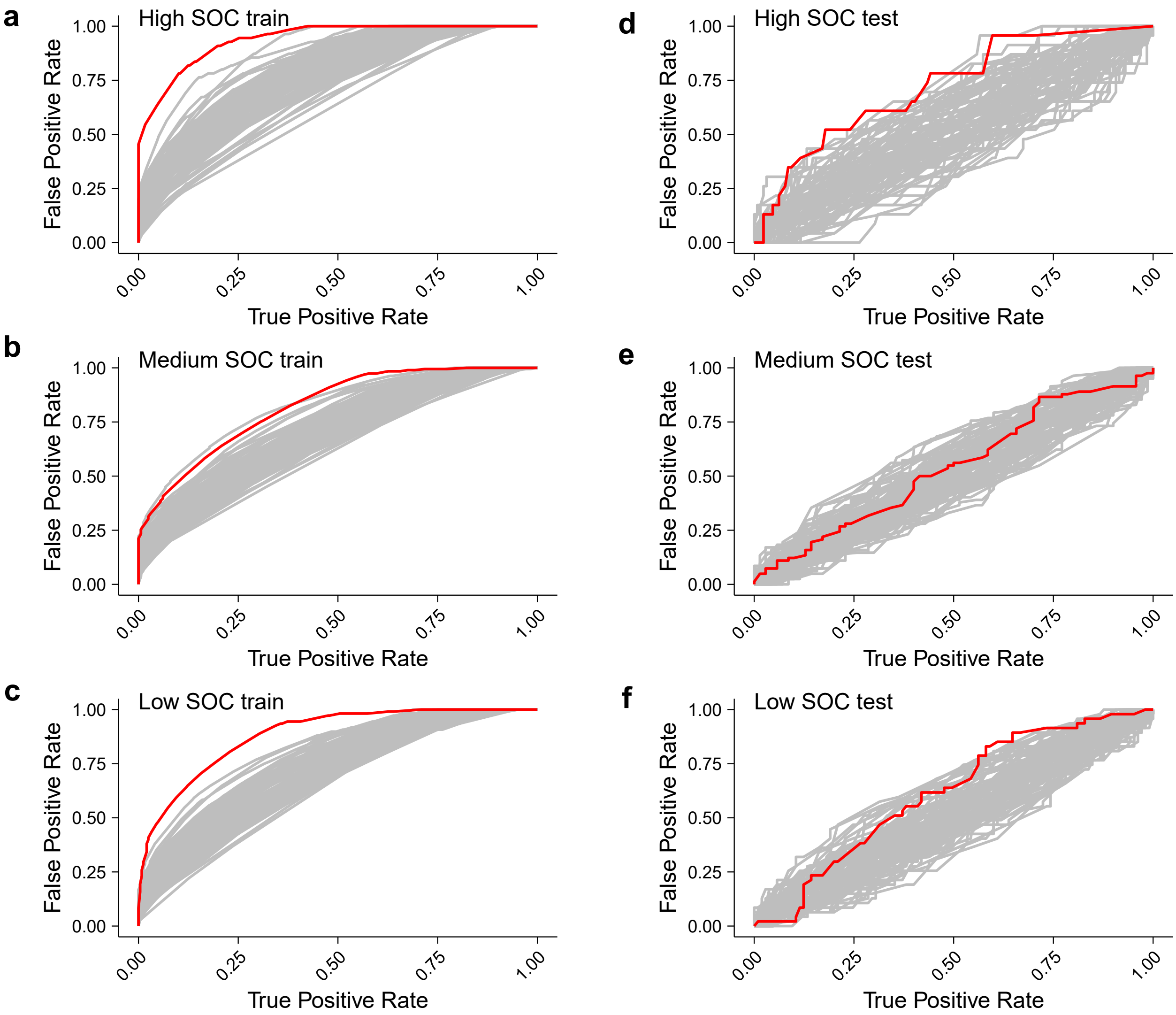

Fig. S4 The performance of metabolite-based Random Forest model for predicting the SOC level.

a-c ROC curves for training data set. **d-f** ROC curves for testing data set. The red curves denote the curves from the selected peak SNPs data set and the grey curves denote the curves from the random SNPs data set.

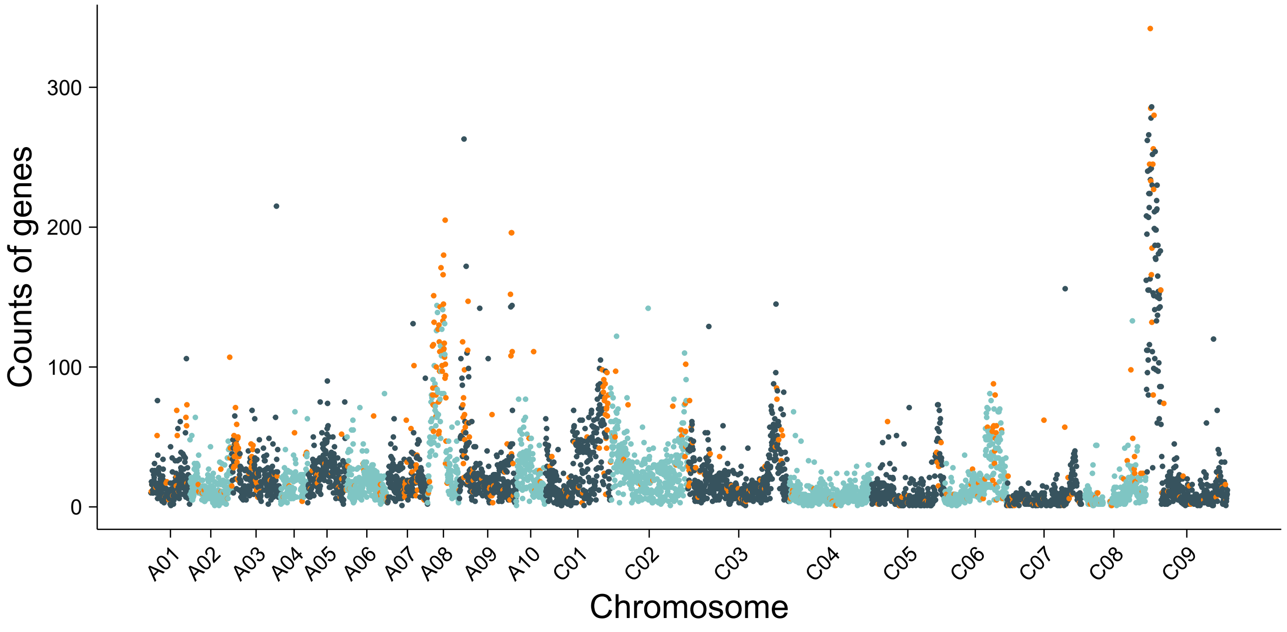

Fig. S5 The genome-wide distribution of eQTLs.

The X-axis represents the physical position of the eQTLs and the Y-axis represents the number of genes associated with the eQTL. The orange point indicates that the eQTL colocalizes with the mQTL detected by mGWAS of SOC-correlated metabolites.

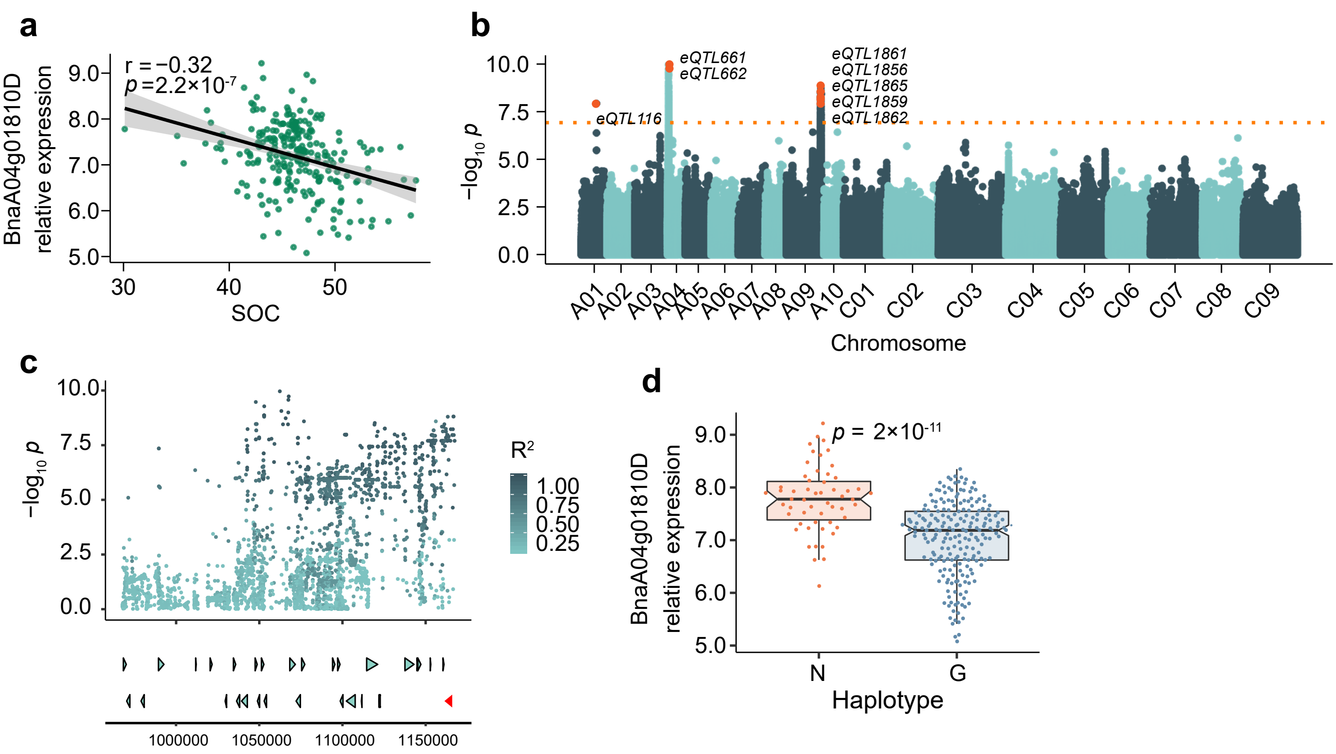

Fig. S6 Genetic analysis of BnaA04g01810D (*SHP1*).

a Correlation analysis between SOC and *SHP1* relative expression. **b** Manhattan plot of eGWAS for *SHP1*. **c** The detailed results of eGWAS for *SHP1*on Chromosome A04. All the dots are centered by the lead SNP marked in **b**. The arrows below the dot plot represent the genes in this location, and BnaA04g01810D is marked in red. **d** *SHP1* relative expression haplotypes analysis.

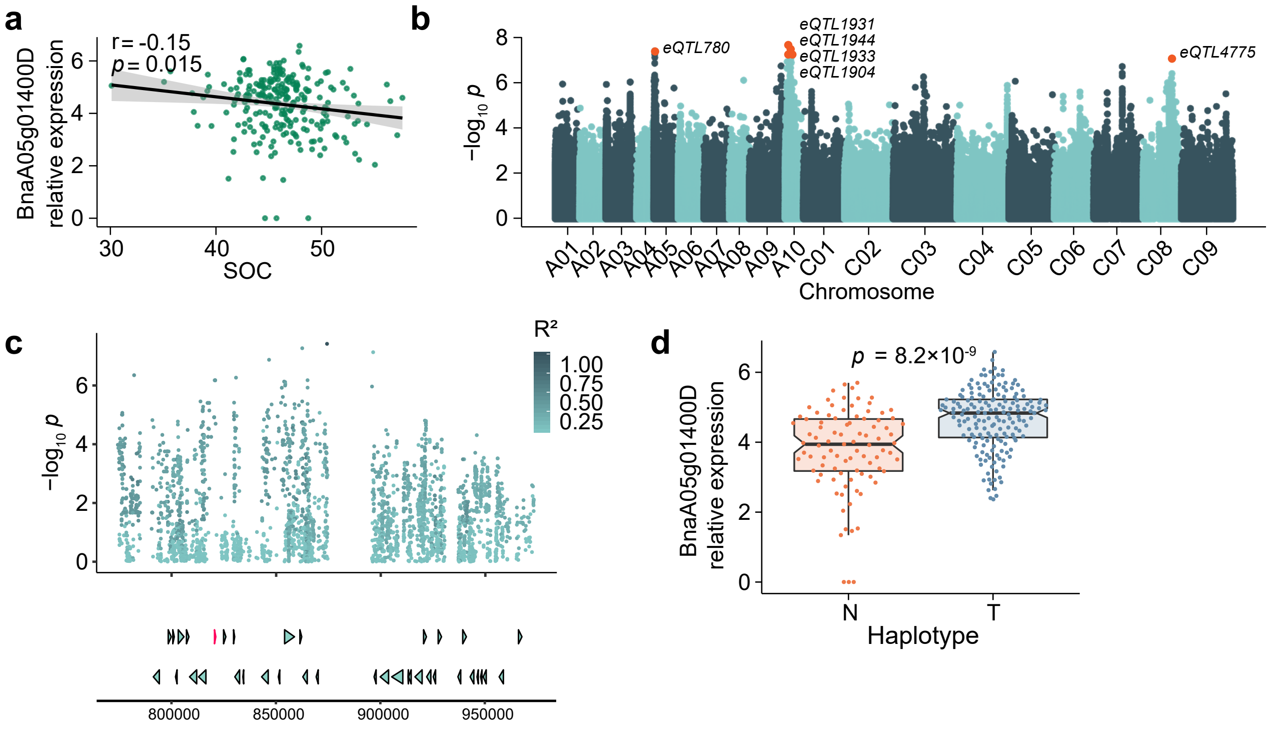

Fig. S7 Genetic analysis of BnaA05g01400D (*CPC*).

a Correlation analysis between SOC and *CPC* relative expression. **b** Manhattan plot of eGWAS for *CPC*. **c** The detailed results of eGWAS for *CPC* on Chromosome A05. All the dots are centered by the lead SNP marked in **b**. The arrows below the dot plot represent the genes in this location, and BnaA05g01400D is marked in red. **d** *CPC* relative expression haplotypes analysis.

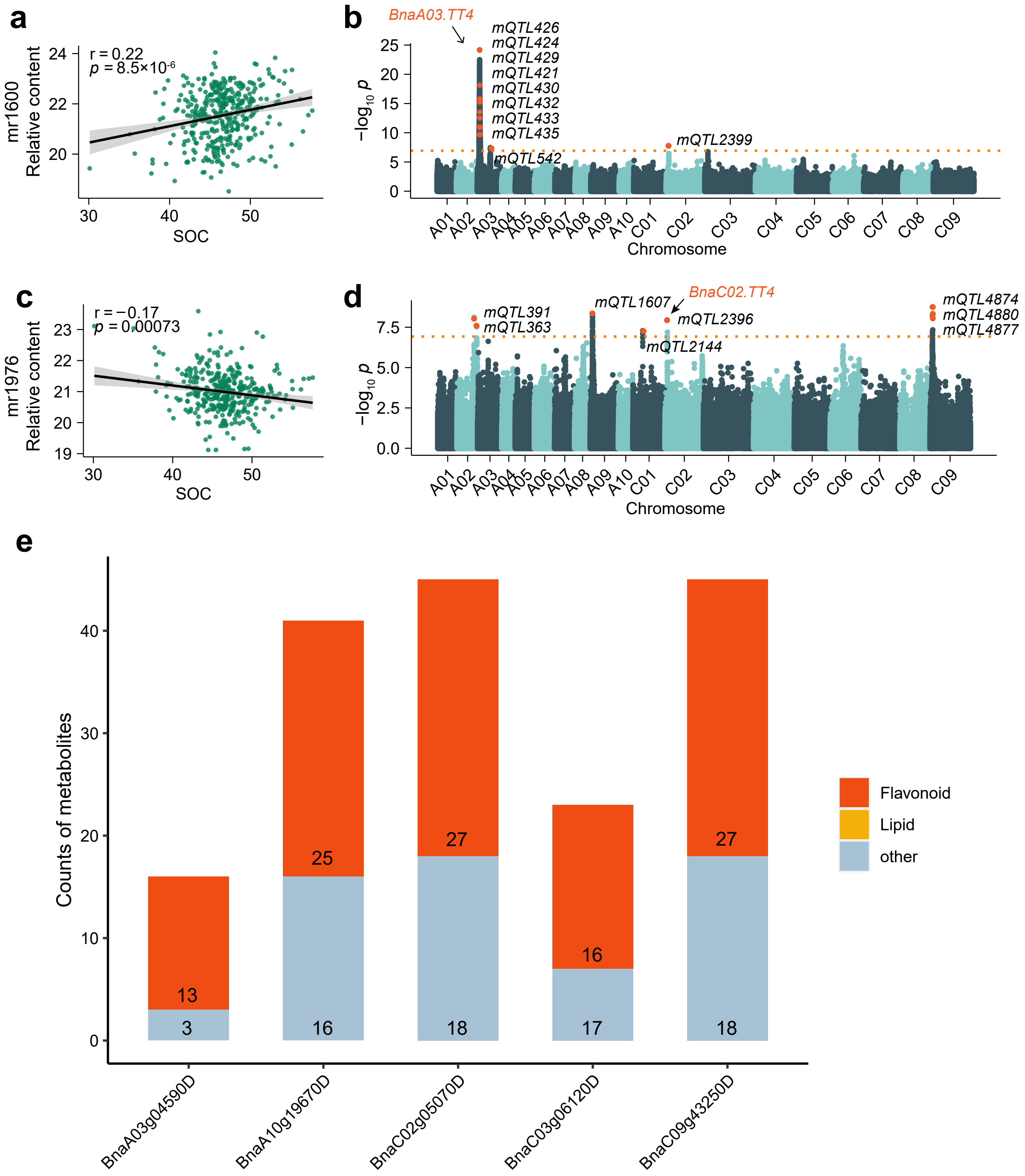

Fig. S8 mGWAS and mTWAS results of *BnaTT4*s.

a Correlation analysis between SOC and mr1600 (Luteolin *C*-sinapoylhexoside). **b** Manhattan plot of mGWAS for mr1600 (2017). **c** Correlation analysis between SOC and mr1976. **d** Manhattan plot of mGWAS for mr1976 (2018). **e** *BnaTT4* homologous gene mTWAS result statistics.

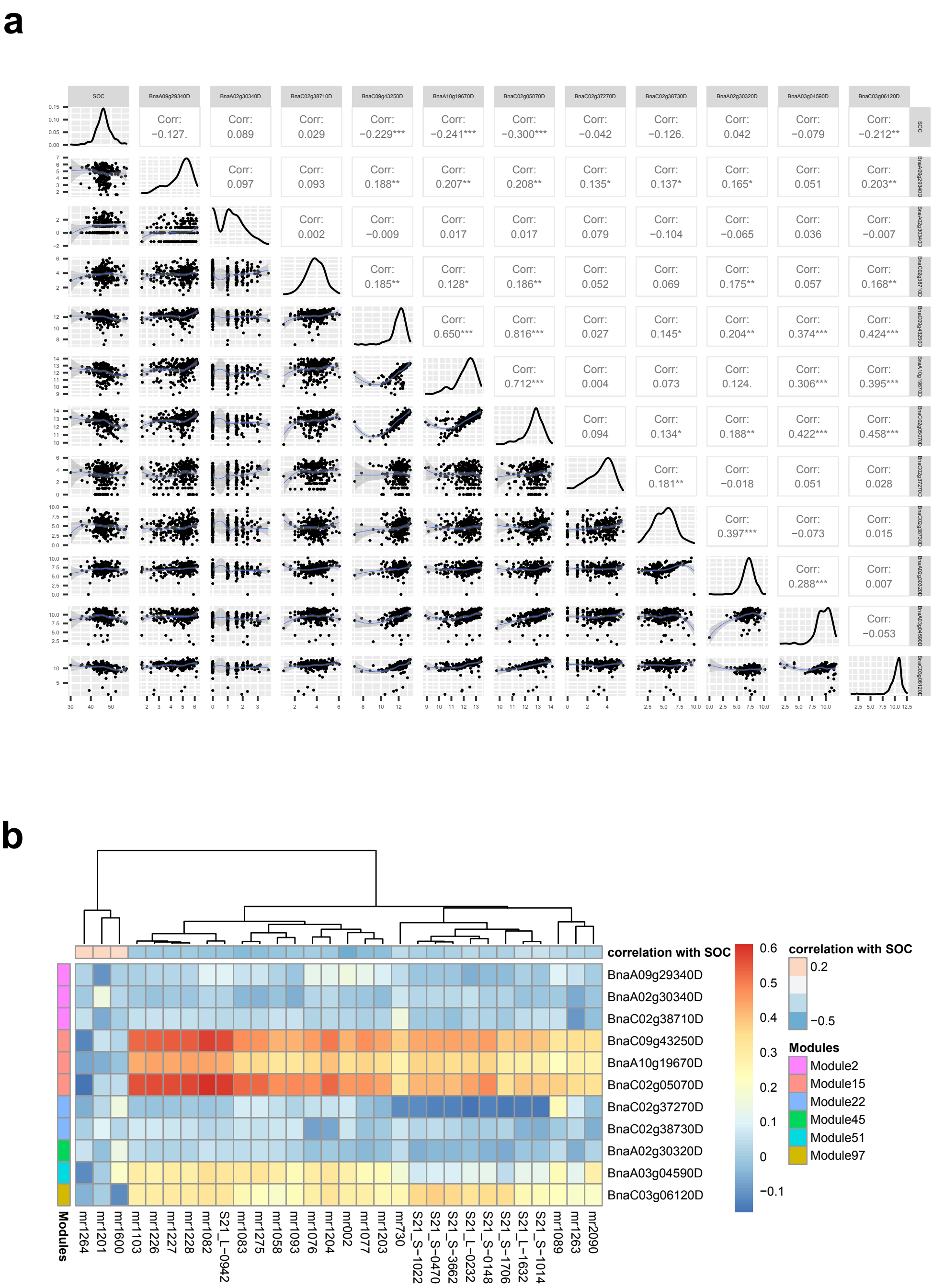

Fig. S9 Correlation analysis of *BnaTT4*s with SOC and SOC-correlated flavonoids.

a Correlation analysis of eleven *BnaTT4*s with SOC. **b** Correlation analysis of eleven *BnaTT4*s with SOC-correlated flavonoids. The fill color of each cell in the heatmap represents the correlation (r) between a *BnaTT4* and a SOC-correlated flavonoid.

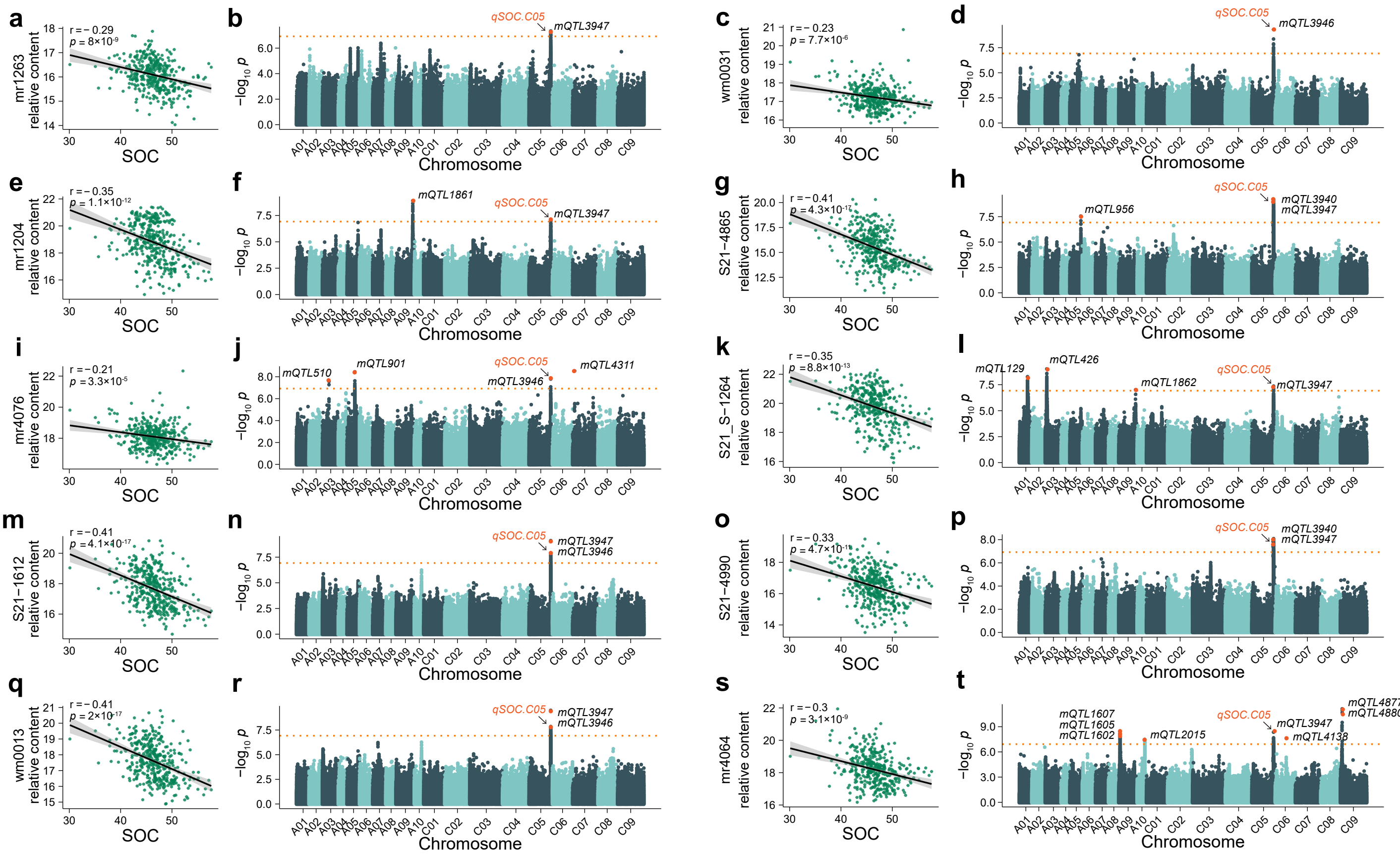

Fig. S10 mGWAS results (2017) related to the loci on chromosome C05 (position = 40,030,860 bp). **a** Correlation analysis between SOC and mr1263 (Naringenin). **b** Manhattan plot of mGWAS for mr1263.**c** Correlation analysis between SOC and wm0031. **d** Manhattan plot of mGWAS for wm0031. **e** Correlation analysis between SOC and mr1204 (Chrysoeriol 7-*O*-hexoside). **f** Manhattan plot of mGWAS for mr1204. **g** Correlation analysis between SOC and S21-4865. **h** Manhattan plot of mGWAS for S21-4865. **i** Correlation analysis between SOC and mr4076 (9(Z),11(E),13(E)-Octadecatrienoic Acid methyl ester). **j** Manhattan plot of mGWAS for mr4076. **k** Correlation analysis between SOC and S21_S-1264. **l** Manhattan plot of mGWAS for S21_S-1264. **m** Correlation analysis between SOC and S21-1612. **n** Manhattan plot of mGWAS for S21-1612. **o** Correlation analysis between SOC and S21-4990. **p** Manhattan plot of mGWAS for S21−4990. **q** Correlation analysis between SOC and wm0013. **r** Manhattan plot of mGWAS for wm0013. **s** Correlation analysis between SOC and mr4064. **t** Manhattan plot of mGWAS for mr4064.

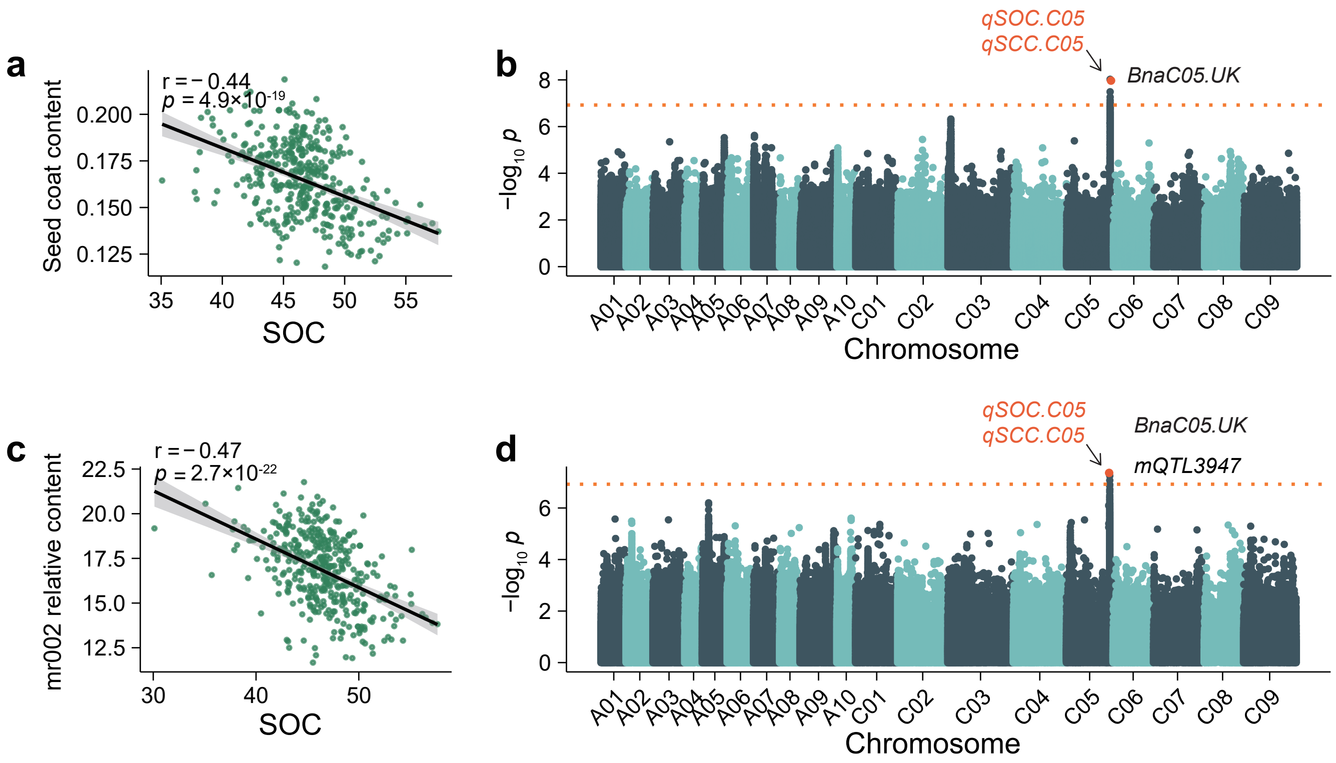

Fig. S11 mGWAS results (2017) related to SCC and mr002 (catechin).

a Correlation analysis between SOC and SCC. **b** Manhattan plot of GWAS for SCC. **c** Correlation analysis between SOC and catechin. **d** Manhattan plot of mGWAS for catechin.

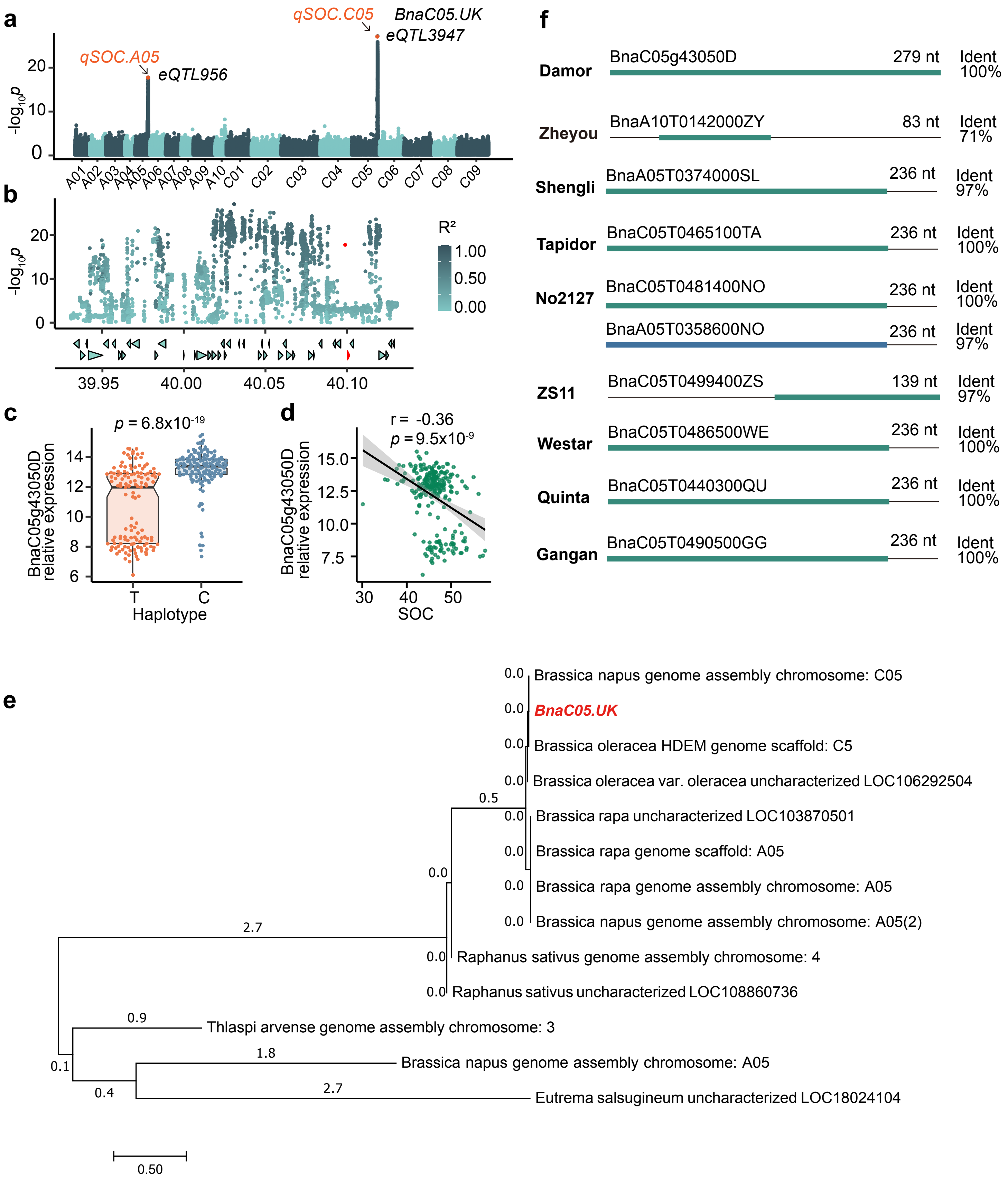

Fig. S12 Functional study of *BnaC05.UK*.

a Manhattan plot of eGWAS results of *BnaC05.UK*. **b** Local Manhattan plot of eGWAS results of *BnaC05.UK* which is marked in red; one variant in the promoter region of *BnaC05.UK* is marked as a red dot. **c** *BnaC05.UK* relative expression haplotypes analysis. **d** Correlation analysis between SOC and *BnaC05.UK* relative expression. **e** Phylogenetic tree of *BnaC05.UK*. **f** Blast results of *BnaC05.UK* in 9 reference genomes.

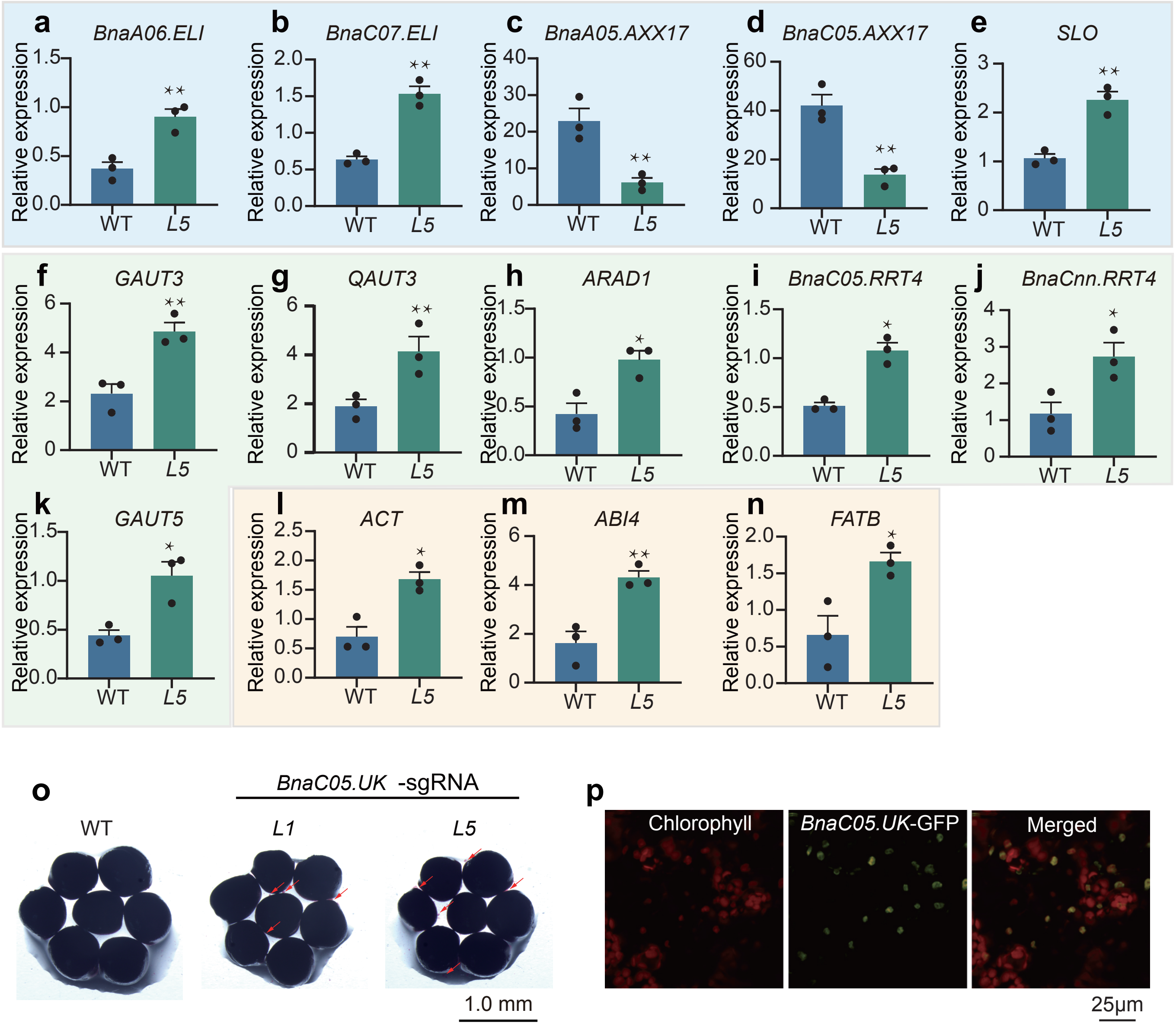

Fig. S13 The relative expression of differentially expressed genes between WT and *L5*.

a-e Chloroplast RNA modification-related genes. **f-k** Mucilage pectin biosynthesis-related genes. **l-n** Oil accumulation-related genes. Values are means ± s.e.m., n = 3, biologically independent samples. Statistical analysis is using Student's t-test (*, *p*-value < 0.05; **, *p*-value < 0.01, two-sided). **o** Mature seed-coat mucilage leakage of *BnaC05.UK* mutants (*L1*, *L5*) and WT. Bars = 1 mm. **p** BnaC05.UK is localized in chloroplast observed in tobacco epidermal cells under confocal microscopy. Bars = 25 mm.

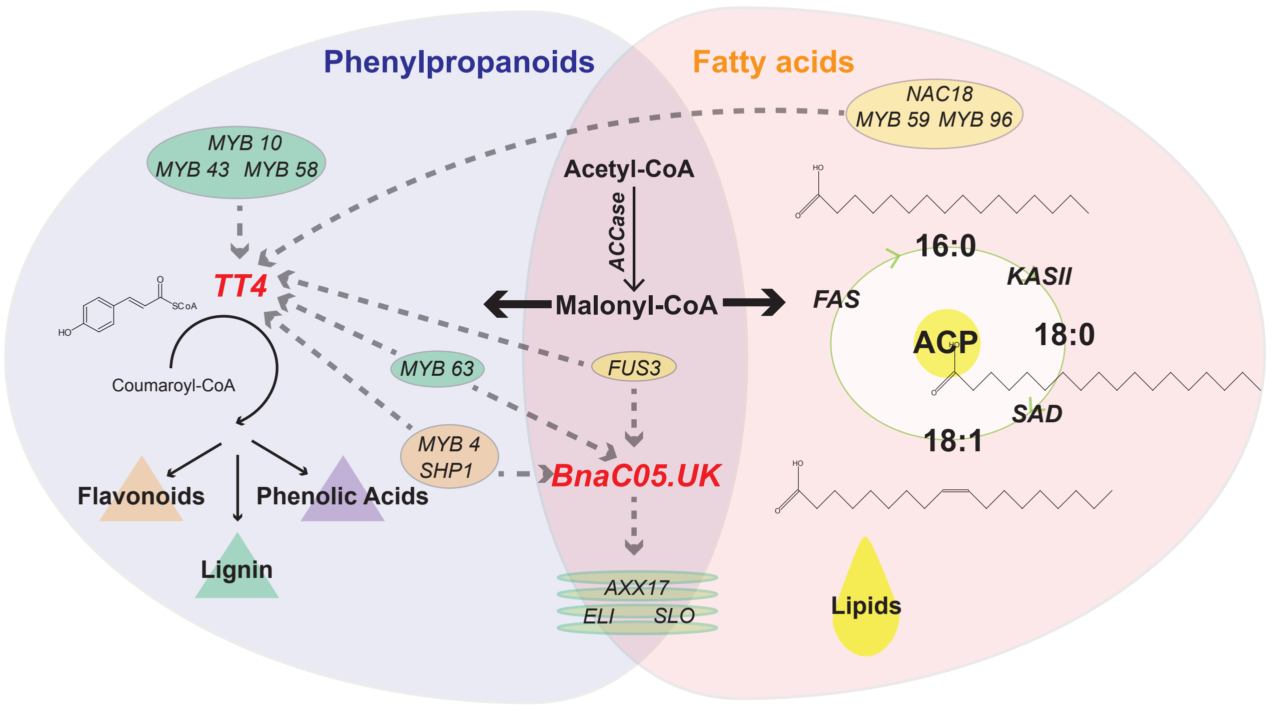

Fig. S14 Phenylpropanoids and fatty acid synthesis relationship and predicted transcriptional regulation of *BnaTT4s* and *BnaC05.UK*.

The ovals present the transcription factors, green represents the lignin synthesis pathway, orange represents the flavonoid synthesis pathway, and yellow represents the fatty acid synthesis pathway. *AXX17*, *ELI* and *SLO* are genes that changed significantly in BnaC05.UK mutant line's transcriptome compared to WT.
