# Supplementary material for: Characterization of novel loci controlling seed oil content in Brassica napus by marker metabolite-based multi-omics analysis
Source: Genome Biol. 2023 Jun 19;24:141. doi: 10.1186/s13059-023-02984-z (PMC10278308; doi:10.1186/s13059-023-02984-z)
Supplement: Supplementary file 3 — Additional file 3. Review History. [file 13059_2023_2984_MOESM3_ESM.docx]

Review History

**First round of review**

**Reviewer 1**

**Were you able to assess all statistics in the manuscript, including the appropriateness of statistical tests used?**

Yes, and I have assessed the statistics in my report.

**Comments to author:**

In this MS, Li et al. used a widely-targeted metabolomics analysis in a natural population of B. napus. They found the Seed oil content (SOC) is significantly affected by the flavonoid metabolism in. Three candidate genes (BnaA03.TT4, BnaC02.TT4 and BnaC05.UK) were revealed to be associated with SOC through regulating flavonoid metabolism. Generally, this MS is well written and easy to read. The experiments were nicely conducted. I only have following comments:

Major comments:

1. Please specify in the text, among the 2173 metabolites, how many metabolites were detected in each metabolic family (flavonoids, fatty acid, terpenoids, alkaloids et al.). In my experience, the contents of the metabolic library would significantly affect the conclusion of association studies. For example, if the majority of the library is flavonoids, the major metabolites determined by association studies are very likely to be flavonoids. The authors need to avoid this bias.

2. This MS, in my opinion, can be a good resource for the plant research society. To improve the accessibility of the data. Please provide the full compound annotation and family classification to table S2. Also, please provide the table for the contents of all the metabolites in all the samples. By doing this, the readers can double-check the conclusion of this MS, as well as use the data for their studies.

3. To me, |r| > 0.2 is too low for a significant association. Even the higher |r| > 0.35 is still too low. Normally I would use |r| > 0.6 (or at least 0.4). If the authors used |r|>0.4, how many metabolites remained? If the metabolites in this MS remain at |r|>0.4, I would use this threshold. It doesn't matter how many metabolites are remaining, the accuracy of the key metabolites is more important.

4. Please provide a global metabolic map illustrating the relationship between SOC and flavonoids as figure 7. And highlight the relationship between SOC and flavonoid in the discussion section. Thus, the readers can better understand the negative correlation between SOC and flavonoids.

5. Personally, based on the conclusion from this MS, I'm more interested in the regulation of BnaTT4s and BnaC05.UK genes. Are there any transcription factors regulating these key genes? I suspect these TFs can be better candidates for gene editing and breeding design.

Other comments:

1、 Line 414, please directly describe, rather than citing the previous paper, the detailed methodology for metabolic profiling.

2、 To support the key conclusion, for the key metabolites in this MS (such as the key SOCs and key flavonoids), please provide mass spectrum as supplemental figures.

3、 Please verify the gene-editing plants. Also, please provide the growth phenotypes of gene-editing plants as supplemental figures. Any growth or yield penalties?

**Reviewer 2**

**Were you able to assess all statistics in the manuscript, including the appropriateness of statistical tests used?**

Yes, and I have assessed the statistics in my report.

**Comments to author:**

Before the submission of this manuscript, this journal had received similar manuscript about gene identification of oil content biosynthesis in rapeseed by using genome, transcriptome and metabolome. In addition, there are also some strange analysis methods such as eQTL. However, more efforts have been devoted to the functional verification of the two genes.

(1) When identifying the specific marker metabolites of SOC, it was found that most of them were flavonoids, while only a few were lipids. Please describe the internal relationship between flavonoids and oil content synthesis? Is it related to the extraction method mentioned later?

Therefore, I can understand that BnaTT4 regulates flavonoid, but I cannot understand the regulation of SOC although the authors provide some evidences by using CRISPR/Cas technology.

(2) Line130-139, this paper carried out WGCNA analysis. The analysis showed that the genes of Module15 related to SOC were mainly concentrated in flavonoids and long-chain fatty-acyl-CoA metabolic processes. Is there any gene involved in oil content that was previously reported?

(3) Line162-165, I didn't understand this sentence, because above this sentence, you are talking about molecular marker assisted breeding but gene discovery.

(4) Line168-171, I also think there is a problem with the calculation method of eQTLs. How can SNPs be divided into eQTLs? Doesn't eQTLs reflect the regulatory relationship between SNP and gene expression? I feel that logic is chaotic.

(5) Line183-191, is there any basic hypothesis about how the genes controlling lignin synthesis participate in the regulation of oil content.

**Authors Response**

**Point-by-point responses to the reviewers’ comments:**

Reviewer #1:

In this MS, Li et al. used a widely-targeted metabolomics analysis in a natural population of B. napus. They found the Seed oil content (SOC) is significantly affected by the flavonoid metabolism in. Three candidate genes (BnaA03.TT4, BnaC02.TT4 and BnaC05.UK) were revealed to be associated with SOC through regulating flavonoid metabolism. Generally, this MS is well written and easy to read. The experiments were nicely conducted. I only have following comments:

Major comments:

1. Please specify in the text, among the 2173 metabolites, how many metabolites were detected in each metabolic family (flavonoids, fatty acid, terpenoids, alkaloids et al.). In my experience, the contents of the metabolic library would significantly affect the conclusion of association studies. For example, if the majority of the library is flavonoids, the major metabolites determined by association studies are very likely to be flavonoids. The authors need to avoid this bias.

*Response: Thank you for your suggestion. We have updated the number of different types of metabolites in Table S6. We also explained our unbiased results by using the hypergeometric test, detailed information is listed in Additional file 1: Table S6, and described the results in Line 90-92 and Line 109-112.*

2. This MS, in my opinion, can be a good resource for the plant research society. To improve the accessibility of the data. Please provide the full compound annotation and family classification to table S2. Also, please provide the table for the contents of all the metabolites in all the samples. By doing this, the readers can double-check the conclusion of this MS, as well as use the data for their studies.

*Response: Thank you for your suggestion. We have provided the full compound annotation and family classification to Additional file 1: Table S2. We have also added contents of all the metabolites in all the samples to Additional file 1: Table S3 and Table S4, and described the results in Line 94-95.*

3. To me, |r| > 0.2 is too low for a significant association. Even the higher |r| > 0.35 is still too low. Normally I would use |r| > 0.6 (or at least 0.4). If the authors used |r|>0.4, how many metabolites remained? If the metabolites in this MS remain at |r|>0.4, I would use this threshold. It doesn't matter how many metabolites are remaining, the accuracy of the key metabolites is more important.

*Response: Thank you for your suggestion. As we listed in the Additional file 1: Table S2, if we set the bar at |r|=0.4, 7 metabolites remained. This study used a large population and the natural variations in metabolites and SOC trait can be controlled by complex factors (multiple loci, genes and networks). |r| > 0.2 is acceptable in this study and allow us to identify reasonable number of marker metabolites. We couldn’t agree more with your opinion that the accuracy of the marker metabolites is more important. For example, mr002 (catechin r=-0.47) is changed significantly in both BnaTT4s (Fig 5b) and BnaC05.UK (Fig 6e) mutants. This metabolite is an absolute marker for SOC of B. napus. However, B. napus seed is occupied with over 40% oil in modern inbreeds. As we all know that secondary metabolites present the characteristic of variety and minority[3]. Therefore, only several key metabolites content changes might not cause a significant shift in SOC. Based on our results, 27 flavonoids are associated with BnaTT4s (Additional file 2: Fig S8) and 16 flavonoids are associated with BnaC05.UK (Additional file 1: Table S8). It’s obvious that the family of flavonoids is strongly correlated with SOC. Rather than call out for certain marker metabolites, the deep-seated mechanism is the balance of fatty acids and flavonoid synthesis. Last but not least, as for the |r| value that we used for a significant association, we calculated that even with our lowest correlated metabolite, the correlation is significant (Additional file 1: Table S5). Similar research methods have been proved to be effective [4-6].*

*ID Oil_Metabolite17_r Oil_Metabolite17_pvalue*

*mr563 -0.202245865 6.27969E-05*

*S21_S-3814 0.202206149 6.30103E-05*

4. Please provide a global metabolic map illustrating the relationship between SOC and flavonoids as figure 7. And highlight the relationship between SOC and flavonoid in the discussion section. Thus, the readers can better understand the negative correlation between SOC and flavonoids.

*Response: Thank you for your suggestion. For a global metabolic map, do you mean build metabolism pathways involving SOC and flavonoid synthesis and show their relationship between flavonoid metabolites and SOC? Because of the complexity of flavonoid metabolism, we provide a simplified global metabolic map illustrating the relationship between SOC and flavonoids as Additional file 2: Fig S14 and highlight the relationship between SOC and flavonoid in the discussion section in Line 367-383. In addition, we also include the potential transcription factors regulating BnaTT4s and BnaC05.UK genes in Fig S14.*

5. Personally, based on the conclusion from this MS, I'm more interested in the regulation of BnaTT4s and BnaC05.UK genes. Are there any transcription factors regulating these key genes? I suspect these TFs can be better candidates for gene editing and breeding design.

*Response: Thank you for your suggestion. We have predicted the potential transcription factors regulating BnaTT4s and BnaC05.UK genes by multi-omics analysis shown in Additional file 2: Fig S17, we also believe that these TFs can be candidates for future study.*

Other comments:

1、 Line 414, please directly describe, rather than citing the previous paper, the detailed methodology for metabolic profiling.

*Response: Thank you for your suggestion. We have added more detail on metabolic profiling methodology in Line 438-445.*

2、 To support the key conclusion, for the key metabolites in this MS (such as the key SOCs and key flavonoids), please provide mass spectrum as supplemental figures.

*Response: Thank you for your suggestion. We have provided key metabolites’ mass spectrum in Additional file 2: Fig S1.*

3、 Please verify the gene-editing plants. Also, please provide the growth phenotypes of gene-editing plants as supplemental figures. Any growth or yield penalties?

*Response: Thank you for your suggestion. We have provided growth phenotypes of gene-editing plants, except for BnaTT4s mutants their thousand seed weight is significantly decreased, we haven’t observed any yield penalties (Additional file 1: Table S15-S16).*

Reviewer #2:

Before the submission of this manuscript, this journal had received similar manuscript about gene identification of oil content biosynthesis in rapeseed by using genome, transcriptome and metabolome. In addition, there are also some strange analysis methods such as eQTL. However, more efforts have been devoted to the functional verification of the two genes.

*Response: Thank you for your suggestion. It’s unfortunate that we couldn’t find any study published in Genome Biology (maybe it is not accepted) you mentioned using genome, transcriptome, and metabolome for gene identification of oil content biosynthesis in B. napus. In our previous studies, we have reported that using GWAS and TWAS, we discovered casual genes controlling seed oil content [1] and seed coat content [2], and it’s been described and discussed in our study (Fig 3, Additional file 2: Fig S3, S10, S11). Our previous studies do not have metabolome data at population scale and in this study we use metabolome and marker metabolites for more in-depth mining of SOC-related loci and genes.*

(1) When identifying the specific marker metabolites of SOC, it was found that most of them were flavonoids, while only a few were lipids. Please describe the internal relationship between flavonoids and oil content synthesis? Is it related to the extraction method mentioned later?

*Response: Yes, it is somehow related to the extraction method which is discussed in the manuscript. Our extraction method, which is using 70% methanol to extract metabolites. The metabolites obtained in this study are mainly highly polar substances. So, we explored the relationship between non-lipid and seed oil content. We found that flavonoids as a represent metabolic family are highly correlated with seed oil content. As other studies showed, there is a substrate competition relationship between flavonoids and fatty acid synthesis[7-9].*

Therefore, I can understand that BnaTT4 regulates flavonoid, but I cannot understand the regulation of SOC although the authors provide some evidences by using CRISPR/Cas technology.

*Response: Based on our multi-omics data, we provided evidence that BnaTT4 genes are significantly associated with flavonoids and correlated with B. napus SOC (Additional file 1: Table S13 and Additional file 2: Fig S8). BnaTT4 is connected with SOC by the bridge of metabolite markers (BnaTT4-flavonoids-SOC). The significant decreases of BnaTT4 mutants seed coat content (Fig 5g) should be directly impacting the SOC since the ratio of embryo (storing oil) is increased. And it is also possible that there is carbon source shifting within the mutant seeds. On the other hand, Xuan et al. have published experimental data about TT4 mutant, and its influence on lipid synthesis in Arabidopsis. Specifically, in the TT4 mutant, the plant improved the utilization of soluble sugar during seed development, activated transcription factors related to glycolysis and fatty acid synthesis such as WRI1、ABI3, etc., and enhanced the accumulation of auxin[10]. Finally, as we predicted the transcriptional factors regulating BnaTT4s, we found that several fatty acids related transcriptional factors could affect the expression of BnaTT4s (Additional file 2: Fig S17).*

(2) Line130-139, this paper carried out WGCNA analysis. The analysis showed that the genes of Module15 related to SOC were mainly concentrated in flavonoids and long-chain fatty-acyl-CoA metabolic processes. Is there any gene involved in oil content that was previously reported?

*Response: Yes, there are. As we listed in Additional file 1: Table S13, not only we clustered co-expression genes by WGCNA, but we analyzed possible associations of these genes among metabolome, genome, and transcriptome. For example, BnaA07g17030D (AT3G56700) encodes a Fatty acyl-CoA reductase 6 (FAR6) in the chloroplast. It is located by two metabolites (mr1263 and S21_S-5574) by mGWAS, and has two eQTLs eQTL1317 (29 genes), eQTL1320 (32 genes). And it also associates with 34 metabolites in mTWAS. AtFAR6 preferentially uses 16 carbon acyl-chains as substrates and produces predominantly fatty alcohols[11]. Similarly, BnaA09g31580D (AT3G50920), encodes a phosphatidic acid phosphatase (LPPε1) that can be detected in chloroplast membrane fractions. It is located by 7 metabolites (S21_L-2819, mr1058, S21_L-1632, S21_L-3903, S21_S-1014, mr1330, mr730), and has three eQTLs eQTL134 (22 genes), eQTL1337 (30 genes), eQTL1633 (99 genes). And it also associates with S21_S-3114 in mTWAS. Similarly, Lipid phosphate phosphatase ε1 (BnaA09g31580D) can affect pollen growth, ER phospholipid biosynthesis, and triacylglycerol biosynthesis[12]. And also 1-acylglycerol-3-phosphate O-acyltransferase (BnaC07g38760D)[13, 14], Phospholipase A1-IIdelta (BnaC04g52540D)[15], Phospholipid-transporting ATPase 10 (BnaA02g27550D)[16], Probable fatty acyl-CoA reductase 4 (BnaC03g53920D)[17, 18], etc.*

(3) Line162-165, I didn't understand this sentence, because above this sentence, you are talking about molecular marker assisted breeding but gene discovery.

*Response: Thank you for your suggestion. We have rephrased the sentence for better logic in Line 168-169.*

(4) Line168-171, I also think there is a problem with the calculation method of eQTLs. How can SNPs be divided into eQTLs? Doesn't eQTLs reflect the regulatory relationship between SNP and gene expression? I feel that logic is chaotic.

*Response: Thank you for your suggestion. Yes, it does. As you expect, eQTLs reflect the regulatory relationship between SNP and gene expression. In our study, we acquired 117,782 lead SNPs from 7,316 selected genes. Each SNP represents the regulatory relationship between the SNP and gene expression correspondingly. Generally, if the physical distances of two or more SNPs are within 100 kb in B. napus, they are considered as one QTL or in this case an eQTL. Therefore, we clustered them into 5,242 eQTLs according to their physical distance for better understanding, like mQTLs. Similar analyses have been proved effective[19-21]. We also rephrased the sentence for better understanding in Lines 173-174.*

(5) Line183-191, is there any basic hypothesis about how the genes controlling lignin synthesis participate in the regulation of oil content.

*Response: Yes, there is. Ehlers et al. reported that SHP1 and SHP2 interact with Transparent Testa 16 (TT16), and they are essential for seed coat development, and shp1 and shp2 mutants have changed fatty acid components[22]. Based on their study and its important roles are in the lignification of dehiscence zone cells in plants[23] and ovule development[24]. We hypothesize that SHP1, SHP2, and TT16 are participants in seed coat cell formation and lignification, also in ovule development. The disruption of SHP1 & SHP2 or TT16 will cause embryonic malformation. Thus, the altered carbon partitioning may result in fatty acid component change and even SOC alteration.*

*Reference:*

*1. Tang S, Zhao H, Lu S, Yu L, Zhang G, Zhang Y, Yang QY, Zhou Y, Wang X, Ma W, et al: Genome- and transcriptome-wide association studies provide insights into the genetic basis of natural variation of seed oil content in Brassica napus. Mol Plant 2021, 14:470-487.*

*2. Zhang Y, Zhang H, Zhao H, Xia Y, Zheng X, Fan R, Tan Z, Duan C, Fu Y, Li L, et al: Multi-omics analysis dissects the genetic architecture of seed coat content in Brassica napus. Genome Biol 2022, 23:86.*

*3. Fernie AR, Trethewey RN, Krotzky AJ, Willmitzer L: Metabolite profiling: from diagnostics to systems biology. Nat Rev Mol Cell Biol 2004, 5:763-769.*

*4. Wen W, Li D, Li X, Gao Y, Li W, Li H, Liu J, Liu H, Chen W, Luo J, Yan J: Metabolome-based genome-wide association study of maize kernel leads to novel biochemical insights. Nat Commun 2014, 5:3438.*

*5. Shi T, Zhu A, Jia J, Hu X, Chen J, Liu W, Ren X, Sun D, Fernie AR, Cui F, Chen W: Metabolomics analysis and metabolite-agronomic trait associations using kernels of wheat (Triticum aestivum) recombinant inbred lines. Plant J 2020, 103:279-292.*

*6. Wei J, Wang A, Li R, Qu H, Jia Z: Metabolome-wide association studies for agronomic traits of rice. Heredity (Edinb) 2018, 120:342-355.*

*7. Zhai Y, Yu K, Cai S, Hu L: Targeted mutagenesis of BnTT8 homologs controls yellow seed coat development for effective oil production in Brassica napus L. Plant Biotechnol J 2020, 18:1153-1168.*

*8. Xie T, Chen X, Guo T, Rong H, Chen Z, Sun Q, Batley J, Jiang J, Wang Y: Targeted Knockout of BnTT2 Homologues for Yellow-Seeded Brassica napus with Reduced Flavonoids and Improved Fatty Acid Composition. J Agric Food Chem 2020, 68:5676-5690.*

*9. Zhang Y, Zhang H, Zhao H, Xia Y, Zheng X, Fan R, Tan Z, Duan C, Fu Y, Li L, et al: Multi-omics analysis dissects the genetic architecture of seed coat content in Brassica napus. Genome Biol 2022, 23:86.*

*10. Xuan L, Zhang C, Yan T, Wu D, Hussain N, Li Z, Chen M, Pan J, Jiang L: TRANSPARENT TESTA 4-mediated flavonoids negatively affect embryonic fatty acid biosynthesis in Arabidopsis. Plant Cell Environ 2018, 41:2773-2790.*

*11. Doan TT, Domergue F, Fournier AE, Vishwanath SJ, Rowland O, Moreau P, Wood CC, Carlsson AS, Hamberg M, Hofvander P: Biochemical characterization of a chloroplast localized fatty acid reductase from Arabidopsis thaliana. Biochim Biophys Acta 2012, 1821:1244-1255.*

*12. Nguyen VC, Nakamura Y: Distinctly localized lipid phosphate phosphatases mediate endoplasmic reticulum glycerolipid metabolism in Arabidopsis. Plant Cell 2023.*

*13. Ghosh AK, Chauhan N, Rajakumari S, Daum G, Rajasekharan R: At4g24160, a soluble acyl-coenzyme A-dependent lysophosphatidic acid acyltransferase. Plant Physiol 2009, 151:869-881.*

*14. James CN, Horn PJ, Case CR, Gidda SK, Zhang D, Mullen RT, Dyer JM, Anderson RG, Chapman KD: Disruption of the Arabidopsis CGI-58 homologue produces Chanarin-Dorfman-like lipid droplet accumulation in plants. Proc Natl Acad Sci U S A 2010, 107:17833-17838.*

*15. Lo M, Taylor C, Wang L, Nowack L, Wang TW, Thompson J: Characterization of an ultraviolet B-induced lipase in Arabidopsis. Plant Physiol 2004, 135:947-958.*

*16. Axelsen KB, Palmgren MG: Inventory of the superfamily of P-type ion pumps in Arabidopsis. Plant Physiol 2001, 126:696-706.*

*17. Domergue F, Vishwanath SJ, Joubès J, Ono J, Lee JA, Bourdon M, Alhattab R, Lowe C, Pascal S, Lessire R, Rowland O: Three Arabidopsis fatty acyl-coenzyme A reductases, FAR1, FAR4, and FAR5, generate primary fatty alcohols associated with suberin deposition. Plant Physiol 2010, 153:1539-1554.*

*18. Kosma DK, Molina I, Ohlrogge JB, Pollard M: Identification of an Arabidopsis fatty alcohol:caffeoyl-Coenzyme A acyltransferase required for the synthesis of alkyl hydroxycinnamates in root waxes. Plant Physiol 2012, 160:237-248.*

*19. Tan Z, Peng Y, Xiong Y, Xiong F, Zhang Y, Guo N, Tu Z, Zong Z, Wu X, Ye J, et al: Comprehensive transcriptional variability analysis reveals gene networks regulating seed oil content of Brassica napus. Genome Biol 2022, 23:233.*

*20. Liu S, Li C, Wang H, Wang S, Yang S, Liu X, Yan J, Li B, Beatty M, Zastrow-Hayes G, et al: Mapping regulatory variants controlling gene expression in drought response and tolerance in maize. Genome Biol 2020, 21:163.*

*21. Ma Y, Min L, Wang J, Li Y, Wu Y, Hu Q, Ding Y, Wang M, Liang Y, Gong Z, et al: A combination of genome-wide and transcriptome-wide association studies reveals genetic elements leading to male sterility during high temperature stress in cotton. New Phytol 2021, 231:165-181.*

*22. Ehlers K, Bhide AS, Tekleyohans DG, Wittkop B, Snowdon RJ, Becker A: The MADS Box Genes ABS, SHP1, and SHP2 Are Essential for the Coordination of Cell Divisions in Ovule and Seed Coat Development and for Endosperm Formation in Arabidopsis thaliana. PLoS One 2016, 11:e0165075.*

*23. Liljegren SJ, Ditta GS, Eshed Y, Savidge B, Bowman JL, Yanofsky MF: SHATTERPROOF MADS-box genes control seed dispersal in Arabidopsis. Nature 2000, 404:766-770.*

*24. Brambilla V, Battaglia R, Colombo M, Masiero S, Bencivenga S, Kater MM, Colombo L: Genetic and Molecular Interactions between BELL1 and MADS Box Factors Support Ovule Development in Arabidopsis. Plant Cell 2007, 19:2544-2556.*

**Second round of review**

**Reviewer 1**

All my previous comments have been addressed.

**Reviewer 2**

In the author’s response, the author said “It’s unfortunate that we couldn’t find any study published in Genome Biology (maybe it is not accepted) you mentioned using genome, transcriptome, and metabolome for gene identification of oil content biosynthesis in B. napus”. Please cite the paper and compare the results between them, the paper is named as “Comprehensive transcriptional variability analysis reveals gene networks regulating seed oil content of Brassica napus”, and in the paper, the author used the resequencing date of 505 accessions and a total of 583 transcriptomes from two seed development stages to perform eQTLs analysis.

**Authors Response**

**Point-by-point responses to the reviewers’ comments:**

Reviewer #2:

In the author’s response, the author said “It’s unfortunate that we couldn’t find any study published in Genome Biology (maybe it is not accepted) you mentioned using genome, transcriptome, and metabolome for gene identification of oil content biosynthesis in B. napus”. Please cite the paper and compare the results between them, the paper is named as “Comprehensive transcriptional variability analysis reveals gene networks regulating seed oil content of Brassica napus”, and in the paper, the author used the resequencing date of 505 accessions and a total of 583 transcriptomes from two seed development stages to perform eQTLs analysis.

*Response: According to your and reviewer 2’s suggestion, we discussed how the results in this study are related to the findings in the previous studies and we also cited our previous study (Tan et al., Genome Biology 2022). The discussion is in the second paragraph in discussion section (reference 70).*
